# Enhanced production and purification of L-asparaginase from *Bacillus paralicheniformis* AUMC B-516 with potent cytotoxicity against MCF-7 cell lines

**DOI:** 10.1186/s13568-025-01890-w

**Published:** 2025-05-22

**Authors:** Abdullah Abobakr Saleh, Hamdy M. El-Aref, Azza M. Ezzeldin, Rania M. Ewida, Osama A. M. Al-Bedak

**Affiliations:** 1 Molecular Biology Researches & Studies Institute, Assiut University, Assiut, 71511, Egypt; 2 Department of Clinical Pathology and Hematological Malignancies, South Egypt Cancer Institute, Assiut University, Assiut, 71511 Egypt; 3 Department of Genetics, Faculty of Agriculture, Assiut University, Assiut, 71511 Egypt; 4 School of Applied Health Sciences, Badr University Assiut, Assiut, Egypt; 5 Clinical Pathology Department, Faculty of Medicine, Assiut University, Assiut, 71511 Egypt; 6 Food Hygiene, Safety and Technology Department, Faculty of Veterinary Medicine, New Valley University, El-Kharga, 72511 Egypt; 7 Assiut University Mycological Centre, Assiut University, Assiut, 71511 Egypt

**Keywords:** L-asparaginase, *Bacillus*, Cancer cell lines, Characterization, Microbial production, Optimization, Purification

## Abstract

L-asparaginase is an important enzyme used in the pharmaceutical and food industries. Nowadays, bacterial species represent the main source of microbial synthesis for L-asparaginase. But studies aimed at improving production yields and new methods that use various microbes to expand the scope of application for the generated enzyme are also necessary for industrial manufacturing. This study focused on the isolation of *Bacillus paralicheniformis* AUMC B-516 and the optimization of L-asparaginase production under submerged fermentation. The enzyme was purified and characterized, followed by an evaluation of its cytotoxic effects against the MCF-7 human breast cancer cell line. The results revealed potent anticancer activity, highlighting the potential application of the purified enzyme in cancer therapeutics. *Bacillus paralicheniformis* AUMC B-516 was utilized for the biosynthesis of L-asparaginase (116.4 U/mL) after 48 h in the presence of 0.2% glucose and 1.0% L-asparagine at 35 °C and pH 8.0. Two-step chromatography (DEAE-cellulose and Sephacryl S200 HR) achieved a 12-fold purification, resulting in an enzyme specific activity of 4087.6 U/mg. For pure L-asparaginase that contained L-asparagine, the Km and Vmax values were 6.22 × 10^−2^ mM and 120.75 µmol/min, respectively. Quantitative assessment of DNA fragmentation in MCF‑7 cells treated with *B. paralicheniformis* B-516’ pure L-asparaginase was performed (22.2 ± 1.36%) and the drug doxorubicin (23.9 ± 0.93%) were significantly greater than those in the negative control cells (8.9 ± 0.83%). MCF-7 cells treated with 1000, 500, 250, 125, 62.5, or 31.25 µg/mL *B. paralicheniformis* AUMC B-516’ pure L-asparaginase showed considerable cytotoxicity, with an IC_50_ of 49.3 µg/mL. Biochemical analyses revealed significant increases in aspartate aminotransferase (AST) and alanine aminotransferase (ALT) activities, while blood glucose, other electrolyte levels, and indicators of renal function remained unchanged. These findings highlight *B. paralicheniformis* AUMC B-516 as a promising source of L-asparaginase for future biotechnological and pharmaceutical applications.

**Supplementary Information:**

The online version contains supplementary material available at 10.1186/s13568-025-01890-w.

## Introduction

Among various amidases, L-asparaginase (L-asparagine amidohydrolase; EC 3.5.1.1) is an enzyme that facilitates the hydrolysis of L-asparagine into L-aspartic acid and ammonia (Kumar and Verma 2012; Saeed et al. 2020). It is an important enzyme used in the pharmaceutical, biosensor and food industries (Radha et al. 2018) and exhibits potent therapeutic potential when combined with other medications to treat melanosarcoma, reticulosarcoma, lymphocytic leukemia, Hodgkin’s lymphomas, chronic lymphosarcoma, acute myelomonocytic leukemia, acute myelocytic leukemia, and acute lymphoblastic leukemia (Verma et al. 2007; El-Naggar et al. 2014; Ali et al. 2016; Husain et al. 2016; Shi et al. 2017; Lenicek Krleza et al. 2024). Chemotherapy, a common cancer treatment, is associated with harmful side effects on normal cells (Sari et al. 2019). This opens the door to exploring modifications in cancer therapies that aim to reduce these side effects while preserving the treatment’s effectiveness. One such modification involves the use of enzymes, particularly L-asparaginase, an aminohydrolase enzyme (Aisha et al. 2022). Interest in this enzyme arose from its antitumor properties and its potential as an anticancer agent. Cancer cells are particularly vulnerable to L-asparagine depletion because they cannot synthesize it, making it essential for their growth. L-asparagine is essential for the metabolism of cancer cells throughout their growth (Al-Rawi 2017).

To synthesize proteins, neoplastic cells must acquire L-asparagine from the extracellular environment because they are unable to stimulate the synthesis of L-asparagine synthetase. L-asparaginase depletes L-asparagine from plasma (Amena et al. 2010; Fontes et al. 2024). This results in an extremely low concentration of L-asparagine, which disrupts protein synthesis and inhibits cell proliferation in neoplastic cells. L-asparagine synthetase enables normal, noncancerous animal cells to synthesize the required L-asparagine (Asselin and Rizzari 2015). Thus, L-asparaginase is used as an antitumor agent for injecting the enzyme intravenously to lower the concentration of L-asparagine, selectively affecting neoplastic cells dependent on this amino acid (Tong et al. 2013).

Although L-asparaginase formulations have advanced significantly, side effects still exist. Thrombosis, hypersensitivity reactions, allergies, acute hepatic dysfunction, myocardial infarction, acute pancreatitis, and clotting abnormalities are among the common side effects (Homans et al. 1987; Raja et al. 2012; Darnal et al. 2023). Although L-asparaginases from *Escherichia coli* and *E.chrysanthemi* have been widely used in medicine, increasing problems such as hypersensitivity, antigenicity, short half-life, transient blood clearance, and unfavorable l-glutaminase-dependent neurotoxicity necessitate continued research to identify more appropriate substitutes (Nguyen et al. 2018; Radadiya et al. 2020; Sobat et al. 2021). Better sources of the enzyme are being investigated in response to issues with commercial L-asparaginases. Consequently, the objectives of the current work are to produce, purify, and characterize L-asparaginase from a wild strain of *Bacillus paralicheniformis* AUMC B-516. The cytotoxicity of the purified enzyme was assessed both in-vitro in the MCF-7 cell line and in-vivo in mice.

## Materials and methods

### Chemicals and reagents

Kits for DNA extraction, PCR, and RNeasy Mini Kit were purchased from QIAGEN (GmbH—Germany—Hilden, Germany). The diethylaminoethyl cellulose (DEAE-cellulose) anion exchanger was purchased from Angene Chemical (Nanjing, China 210,061). Sephacryl S 200 HR gel was purchased from Sigma‒Aldrich (St. Louis, MO, USA). RPMI 1640 medium was purchased from Gibco (168 Third Avenue, Waltham, MA, USA). Antibiotic‒antimycotic combination was purchased from Capricorn Scientific (Capricorn Scientific GmbH, Auf der Lette 13A, 35085 Ebsdorfergrund, Germany). l-glutamine were purchased from Lonza—Lonza Group (AG, Muenchensteinerstrasse 38, Switzerland). RQ1 RNAse-free DNAse was purchased from Invitrogen (Thermo Fisher Scientific, Carlsbad, CA, USA). RevertAid™ First Strand cDNA Synthesis Kit was purchased from Fermentas (Germany). Absolute ethyl alcohol was purchased from El Nasr Chemical Industries Company (El Nasr for Chemical Industries, Egypt).

### Bacterial isolates

The Assiut University Mycological Centre (AUMC) culture collection yielded one hundred bacterial isolates, which were then tested for the production of L-asparaginase. Textile effluent tainted with azo dyes yielded all of the bacterial isolates.

### Preliminary screening of L-asparaginase activity

Modified M9 medium (1000 mL of distilled water: Na_2_HPO_4_·2H_2_O, 6.0 g; KH_2_PO_4_, 3.0 g; NaCl, 0.5 g; L-asparagine, 10.0 g; 1 mol/L MgSO_4_·7H_2_O, 2.0 mL; 0.1 mol/L CaCl_2_·2H_2_O, 1.0 mL; 2% glucose stock, 10.0 mL; agar, 20.0 g.) (Gulati et al. 1997) was used for the detection of L-asparaginase activity in the tested bacterial isolates. Ten milliliters of M9 medium was poured into sterile Petri dishes (approximately 10 mL per dish), and the dishes were autoclaved at 121 °C for 20 min. The petri dishes were inoculated individually with 50 μL of spore suspension (1.8 × 10^8^ spores/mL) obtained from 24-h-old cultures of the tested bacteria. The generation of a pink color around the bacteria suggested the development of L-asparaginase due to the release of ammonia attributable to L-asparagine degradation. The tested bacteria were categorized as high, moderate, or low L-asparaginase producers according to measurements of the pink zone diameter surrounding the bacterial growth zone.

### Determination of L-asparaginase activity in submerged fermentation (SmF)

The high-L-asparaginase producers selected from the previous screening were cultured in 250 mL Erlenmeyer conical flasks, each containing 50 mL of Modified M9 medium. The initial pH was adjusted to 6.2. The flasks were then separately inoculated with 2 mL of spore suspension (1.8 × 10^8^ spores/mL) obtained from 24-h-old cultures of the tested bacteria. The inoculated flasks were incubated for 7 days at 30 °C under shaking conditions at 150 rpm. After the incubation period, the cell-free supernatant was obtained via centrifugation (10,000 rpm at 4 °C for 10 min) and used as the L-asparaginase source.

### L-asparaginase assay

The enzyme assay was performed via the Nesslerization method (Imada et al. 1973). The reaction mixture contained 0.5 mL of 0.04 M L-asparagine + 0.5 mL of 0.05 M Tris–HCl buffer (pH 6.8) + 0.5 mL of an enzyme filtrate + 0.5 mL of distilled water. The mixture was incubated at 37 °C for 30 min. afterwards, the reaction was stopped by the addition of 0.5 mL of 1.5 M trichloroacetic acid (TCA). A 0.1 mL aliquot of the above mixture was mixed with 0.2 mL of Nessler’s reagent and 3.7 mL of distilled water and kept at 20 °C for 20 min. The absorbance at 480 nm was measured, and the amount of released ammonia was determined via the use of ammonium sulfate as a standard. One unit of L-asparaginase is defined as the amount of enzyme that liberates 1 µmol of ammonia under standard assay conditions according to Eq. (1).

1 $$ \begin{aligned} & {\text{L-asparaginase activity}} \\ & \quad = \frac{{{\text{Absorbance }} \times 1000 \times {\text{ Enzyme DF }}}}{{{\text{Slope }} \times {\text{ M}}.{\text{Wt}}. \times {\text{ Time}}}}{\text{U}}/{\text{mL}} \\ \end{aligned} $$

where DF = dilution factor nzyme and M. Wt. = molecular weight of L-asparagine. 

The concentration of proteins was determined according to the method described by Lowry (Lowry et al. 1951; Waterborg 2009). A standard curve of bovine serum albumin (BSA), ranging from 10 to 500 µg/mL, was used to determine the protein concentration.

### Molecular identification of the potent bacterial isolate

#### DNA isolation, PCR amplification, and DNA sequencing

Genomic DNA was extracted from the bacterial isolate AUMC B-516 via a standard DNA extraction protocol. The procedure involved growing the bacterial culture in nutrient broth (50 mL), and the culture was incubated at 37 °C until reaching the desired growth, followed by the use of a commercial DNA extraction kit, or a phenol‒chloroform extraction method, depending on the strain’s characteristics. The quality and quantity of the extracted DNA were assessed via a spectrophotometer (NanoDrop) and agarose gel electrophoresis (Sambrook et al. 1989). For PCR amplification, the extracted DNA was used as a template to amplify the 16S rRNA gene, which is commonly used for bacterial identification and phylogenetic analysis. The amplification was performed via the universal primers 27F (5′-AGA GTT TGA TCC TGG CTC AG-3′) and 1492R (5′-GGT TAC CTT GTT ACG ACT T-3′), as described by Weisburg et al. (1991). The PCR mixture consisted of a DNA template, primers, dNTPs, buffer, and Taq polymerase. The specific concentrations of each component used in the PCR were: DNA template (100 ng), forward primer (0.3 µM), reverse primer (0.3 µM), dNTPs (200 µM each), Taq polymerase (1.0 U), buffer (1x, containing MgCl₂ 1.5 mM), and water to a final volume of 50 µL. The amplification cycle conditions included initial denaturation at 95 °C for 5 min, followed by 30 cycles of denaturation at 95 °C for 30 s, annealing at 55 °C for 30 s, and extension at 72 °C for 1 min, with a final extension step at 72 °C for 10 min. The amplified product was confirmed through agarose gel electrophoresis, and the expected 16S rRNA gene fragment (approximately 1500 bp) was visualized. Subsequent to successful amplification, the PCR product was purified via a PCR purification kit, and sequenced in both directions via the same primers. DNA sequencing was performed on an automated sequencer, and the obtained sequences were analyzed and compared with those in GenBank via BLAST (Altschul et al. 1990). Phylogenetic analysis was conducted via MEGA software to determine the relationships of AUMC B-516 with closely related bacterial species.

### Phylogenetic analyses

Using the DNASTAR computer program (version 5.05), the contiguous sequence of the bacterial isolate AUMC B-516 used in this investigation was generated. Using MAFFT (Katoh and Standley 2013), all the sequences were aligned via the default settings. BMGE was used to optimize alignment gaps and parsimony uninformative characters (Criscuolo and Gribaldo 2010). MEGA X (version 10.2.6) was used to conduct maximum likelihood (ML) and maximum parsimony (MP) phylogenetic analyses (Kumar et al. 2018). By using 1000 bootstrap replications, the resilience of the most frugal trees was assessed (Felsenstein 1985). Using Modeltest 3.7’s implementation of the Akaike information criterion (AIC), identified GTR + G + I (General Time Reversible with gamma-distributed rates and a proportion of invariant sites) as the best-fit nucleotide substitution model, which was then applied in all ML analyses (Posada and Crandall 1998).

### Optimization of fermentation parameters

Using one factor at time (OFAT), the maximization of L-asparaginase production was studied at various pH values (4–10), nitrogen sources (ammonium chloride, ammonium sulfate, sodium nitrate, sodium nitrite, urea, peptone and yeast extract) at 0.2%, incubation temperatures (25, 30, 35, 40, 45, and 50 °C), and fermentation periods (1–7 days). The control fermentation medium contained 0.2% (w/v) L-asparagine as the sole nitrogen source, against which all other nitrogen supplements were compared. The enzyme assay was carried out as previously described, and the optimum parameters were chosen for enzyme production.

### Amino acid analysis

Amino acid analysis was performed on the ethanol-precipitated fraction to confirm the presence of the target protein and guide subsequent purification steps. A validated standard was included, and the analysis confirmed that the amino acid composition of the sample matched that of the target protein, with no evidence of interference from unwanted proteins. This preliminary analysis provided valuable compositional information for optimizing the purification process. The final purified protein was subjected to additional validation to ensure data accuracy. The amino acid analysis and determination were carried out by the Chromatography Laboratory at the National Research Centre, Giza, Egypt, via the techniques outlined by Campanella et al. (2002), Laurens et al. (2012), and Jajić et al. (2013). After 0.1 g of pure L-asparaginase was mixed with 5.0 mL of water and 5.0 mL of 6 M HCl, the mixture was heated to 120 °C for 24 h before being filtered. In the end, 1.0 mL of the filtrate was added to an Agilent 1260 series HPLC after being dried and suspended in 0.1 M HCl. A pre-column derivatization step was included, samples were automatically mixed in the autosampler with OPA/MPA reagent (0.5 µL, 2 min reaction) followed by FMOC reagent (0.4 µL, 2 min reaction) before drawing 32 µL injection volume. An Eclipse Plus C18 column (4.6 mm × 250 mm; 5 μm diameter) was used for separation. The mobile phase was created by mixing a solvent mixture of acetone, methanol, and water (45:45:10) at a flow rate of 1.5 mL/min with sodium phosphate dibasic/sodium borate buffer (pH 8.2). A linear gradient was used to program the mobile phase in sequential order.

### L-asparaginase purification (dialysis and ethanol precipitation)

After incubation, the cell-free supernatant was recovered by centrifugation at 10,000 rpm for 10 min at 4 °C. Cold absolute ethyl alcohol (− 25 °C) was used to isolate the enzyme at 4 °C. The isolated protein was dissolved in Tris buffer (pH 8.0), dialyzed twice for 2 h at room temperature (cutoffs: 12–14 kD), and then cooled overnight at 4 °C to remove salts and other small molecules. The dialyzed protein was then concentrated at reduced temperature and pressure via a freeze dryer (VirTis, model #6KBTES-55, NY, USA). Ethanol was selected for protein precipitation after preliminary optimization experiments demonstrated its ability to preserve the stability and activity of the target protein while providing effective precipitation under low-temperature conditions.

### Ion exchange column chromatography (DEAE-cellulose)

A glass column was packed with diethylaminoethyl (DEAE) cellulose, an anion exchange resin (50 cm × 2.4 cm; bed volume 100 cm^3^) and activated by 0.5 M NaOH for 60 min. A 5.0 mL sample was placed onto the column after phosphate buffer (100 mM, pH 8.0) had been used to equilibrate it. The enzyme was eluted with 100 mM phosphate buffer (pH 8.0) at NaCl concentrations of 0, 0.1, 0.25, 0.5, 1.0, and 1.5 M. The column flow rate was adjusted to 0.25 mL/min, and the fractions were 5.0 mL in volume. The previously mentioned method was used to measure L-asparaginase activity. The most active fractions (5 mL each) were assayed for L-asparaginase activity and protein concentration, and those with the highest specific activity (U per mg protein) were designated most active, pooled, concentrated and stored for further purification.

### Sephacryl S 200 HR gel filtration column chromatography

In a glass column (50 cm × 2.4 cm; bed volume 100 cm^3^), Sephacryl S 200 HR gel was packed. The protein was eluted via phosphate buffer (100 mM, pH 8.0) after this column was loaded with the concentrated enzyme sample. L-asparaginase activity was evaluated via the abovementioned method in 5.0 mL fractions. The most active fractions were pooled, concentrated, and lyophilized.

### Sodium dodecyl sulfate–polyacrylamide gel electrophoresis (SDS‒PAGE)

A 0.1 g sample of L-asparaginase was dissolved in 100 µL of 20 mM Tris–HCl buffer at pH 7.4 (Invitrogen, USA), which included 4.0% sodium dodecyl sulfate (SDS). A 12% SDS polyacrylamide gel was loaded with the entire cell lysate. Coomassie brilliant blue R-250 was used for protein staining. Once the unbound dye was removed from the gel and the stained proteins were visible as blue bands, the gel was placed into a destaining solution. The gel was subsequently imaged using Quantity One software, version 4.6.2.

### Impact of pH, temperature, metal ions on the activity of pure L-asparaginase

The effect of pH (3.0–11.0) on pure L-asparaginase activity was determined at temperatures of 21, 24, 27, 30, 33, 36, 39, 42, and 45 °C. Enzyme samples were equilibrated for 10 min in 0.1 M buffers prepared to maintain consistent ionic strength: citrate (pH 3–6), phosphate (pH 7–8), or glycine–NaOH (pH 9–11). Reactions were initiated by adding 0.01 g of enzyme powder and 0.01 g of L-asparagine, each dissolved separately in 1.0 mL of the chosen buffer, and incubated for 30 min at the test temperature. The reaction was stopped by adding 2.0 mL of cold 10% Trichloroacetic acid (TCA), and released ammonia was quantified by Nesslerization.

### Effect of metal ions and chelation

Metal ions, such as NaCl, KCl, CaCl_2_, MgSO_4_, MnSO_4_, FeSO_4_, CuSO_4_, ZnSO_4_, CoCl_2_, CdCl_2_, and BaCl_2,_ were examined by adding them to a solution at a concentration of 5 mM. Moreover, enzyme chelation was examined via the use of 5 mM ethylenediaminetetraacetic acid (EDTA). To determine 100% activity, the activity of L-asparaginase was measured under conventional reaction conditions without the presence of metal ions or EDTA. There were three runs of the experiment.

### Determination of K_m_ and V_max_

The Michaelis‒Menten constant (km) and maximum reaction velocity (Vmax) values of the pure L-asparaginase were determined by measuring the enzyme activity at different concentrations of L-asparagine (2–20 mM) via a Lineweaver‒Burk plot (Lineweaver and Burk 1934) according to Eq. (2).

2 $$\frac{1}{\text{v}}=\frac{1}{{V}_{max}}+\frac{{\text{K}}_{\text{m}}}{{V}_{max}} \times \frac{1}{\text{S}}$$

### Molecular docking

#### Protein structure preparation

The amino acid sequence of L-asparaginase from *B. paralicheniformis* AUMC B-516 used in this study (WP_105980685.1) was retrieved from the NCBI protein database. The Robetta server was used to predict the 3D structures of these proteins (Song et al. 2013). AlphaFold v2.0 was then used to refine the predicted structures to obtain high-accuracy 3D models (Jumper et al. 2021). The active sites in the refined models were predicted via the DeepSite server (Jiménez et al. 2017).

#### Ligand structure preparation

The PubChem database (https://pubchem.ncbi.nlm.nih.gov/) provides the structure of L-asparagine (ID 6267) (Kim et al. 2016). With the aid of software, the structure was created via Avogadro v1.2.0 (Hanwell et al. 2012). The force field MMFF94 (Halgren 1996) was used to optimize the geometry.

#### Antigenicity prediction

In accordance with Doytchinova and Flower (Doytchinova and Flower 2007), the protein sequences of L-asparaginase from *B. paralicheniformis* were uploaded to the Vaxijen v2.0 portal to estimate its antigenicity. Supervisory machine learning techniques were employed to train alignment-independent predictors on the basis of the ACC vectors. Vaxijen enabled the categorization of input sequences as antigens or non-antigens according to the predictive model. We employed the standard 0.5 antigenicity cutoff score.

#### Molecular docking

AutoDock Vina (https://vina.scripps.edu/) was used to molecularly dock the produced L-asparagine ligand to the anticipated L-asparaginase structures (Trott and Olson 2010). The whole protein structure was included in the definition of the docking box, and exhaustiveness was set to eight.

### Protein‒protein interactions

#### Protein preparation

The UniProt database (https://www.uniprot.org/) provides the amino acid sequences of the human glutaminase liver isoform (Q9UI32), human glutaminase kidney isoform (O94925), L-asparagine (P08243), and L-asparaginase from *B. paralicheniformis* (WP_105980685.1). DeepSite was used to determine the binding locations (Feinstein and Brylinski 2014).

#### Protein‒protein docking

The HDOCK server (http://hdock.phys.hust.edu.cn/) was utilized for molecular docking (Yan et al. 2017). The amino acid binding sites were identified as restricted docking regions of glutaminase isoforms (receptors). The top 10 poses were produced for each docking run via restricted docking on the basis of prior amino acids and default parameters.

### Cytotoxic activity against human cell lines

#### MTT assay

The Bioassay and Cell Culture Laboratory at the National Research Centre, Giza, Egypt, carried out and analyzed an in-vitro bioassay on human tumor cell lines. Microscopy images were acquired via an Olympus CKX41 microscope with a U-CMAD3 camera (Olympus Corporation, Japan), and the objective lenses used were 40 × with a numerical aperture (N.A.) of 0.75. RPMI 1640 medium containing a 1.0% antibiotic‒antimycotic combination and 1.0% l-glutamine was used (Mosmann 1983). The cells were batch grown for 10 days before being seeded at a concentration of 10 × 10^3^ cells/well in new complete growth medium. To obtain final concentrations of 100, 50, 25, 12.5, 6.25, 3.125, 1.56 and 0.78 µg/mL, the medium was aspirated, fresh medium (without serum) was added, and the cells were cultured either alone (negative control) or with various doses of sample. The medium was aspirated after 48 h of incubation, and 40 µL of MTT salt (2.5 μg/mL) was added to each well. Two hundred microlitres of 10% sodium dodecyl sulphate (SDS) in deionized water were added to each well and incubated overnight at 37 °C to terminate the reaction and dissolve the crystals that had formed. Doxorubicin (100 µg/mL) was used as a positive control (Thabrew et al. 1997; El-Menshawi et al. 2010). Afterwards, a microplate multiwell reader (Bio-Rad Laboratories Inc., model 3350, Hercules, California, USA) was used to measure the absorbance at 595 nm to determine the IC_50_ and IC_90_. The percent change in viability was calculated via Eq. (3).

3 $$ \% {\text{ Cell viability}} = \left[ {\left( {\frac{{{\mathbf{Reading}} {\mathbf{of}} {\mathbf{sample}} }}{{{\mathbf{Reading}} {\mathbf{of}} {\mathbf{negative}} {\mathbf{control}}}}} \right){-}{1}} \right] \times {1}00 $$

#### Apoptosis assay

The apoptotic effect of the Aspergillus L-asparaginase was examined on HCT-116, HepG-2 and PC-3 cells using Annexin V-FITC/PI-PE double-staining assay. Determination of the apoptotic effect of the enzyme was determined by obeying to the manufacturer’s instructions (Altay et al. 2019; Kalın et al. 2022). Accordingly, the cells (3 × 10^5^ cells/well) were planted into a 6-well culture plate and exposed to the 25, 50 and 100 μg/mL of the enzyme for 48 h. After incubation, the collected cells in 1 mL of complete medium including fetal bovine serum (FBS, 1%). Afterward, the cell suspension (100 μL) was mixed with FITC AnnexinV/ propidium iodide and vortexed for 3–5 s. After incubation for 20 min at room temperature and monitored by flow cytometry Analyzer. The flow cytometry results were represented with dot blot graphs. Untreated cells were used as control group cells.

#### Assay for DNA fragmentation (gel electrophoresis laddering)

DNA fragmentation test was performed in accordance with (Yawata et al. 1998)the instructions for the MCF-7 breast cancer cell line. The cell lines were homogenized in 1 mL of RPMI 1640 medium and then subjected to different substances, such as L-asparaginase from B-516 and the drug doxorubicin. The cells were treated with L-asparaginase at a dose of 50 IU/mL for 24 h and then exposed to 1 μM doxorubicin for 24 h. After that, the cells were centrifuged at 800 rpm for 10 min. The cells were then harvested and washed with Dulbecco’s phosphate-buffered saline. For 30 min on ice, the MCF-7 cells were lysed via a lysis mixture that contained 10 mM Tris base (pH 7.4), 150 mM NaCl, 5 mM EDTA, and 0.5% Triton X-100. The lysates were subsequently centrifuged for 20 min at 10,000 rpm. The fragmented DNA was extracted from the supernatant via an equal mixture of neutral phenol, chloroform, and isoamyl alcohol (25: 24: 1). The samples were then subjected to electrophoretic analysis using 2.0% agarose gels stained with 0.1 μg/mL ethidium bromide.

### Procedure for the diphenylamine reaction

In this work, MCF-7 cells were harvested immediately following culture, treated with the medication doxorubicin, and treated with L-asparaginase from *B. paralicheniformis* AUMC B-516. After being lysed in 0.5 mL of lysis buffer containing 10 mM Tris–HCl (pH 8.0), 1.0 mM EDTA, and 0.2% Triton X-100, the cancer cells were centrifuged for 20 min at 4 °C at 10,000 rpm. The pellets were resuspended in 0.5 mL of lysis buffer. 0.5 mL of 25% trichloroacetic acid (TCA) was added to the pellets (P) and supernatants (S), and the mixture was incubated for 24 h at 4 °C. After the cells were centrifuged for 20 min at 10,000 rpm and 4 °C, the pellets were suspended in 80 mL of 5.0% TCA and incubated for 20 min at 83 °C. After that, 160 mL of a diphenylamine (DPA) solution—which contained 150 mg of DPA in 10 mL of glacial acetic acid, 150 mL of sulfuric acid, and 50 mL of acetaldehyde (16 mg/mL)—was added to each cell sample. The samples were then allowed to incubate for 24 h at room temperature (Gibb et al. 1997). The absorbance was measured at 600 nm, and the percentage of fragmented DNA was determined via Eq. (4).

4 $$ \% {\text{ Fragmented DNA}} = \frac{{{\text{OD }}\left( {\text{S}} \right)}}{{{\text{OD }}\left( {\text{S}} \right) + {\text{ OD }}\left( {\text{P}} \right)}} \times { 1}00 $$

where S stands for supernatants, P for pellets, and OD for optical density.

### Analysis of gene expression

Total RNA from the MCF-7 cell line was isolated via an RNeasy Mini Kit in conjunction with a QiagenDNaseI digestion step according to the manufacturer’s instructions. The isolated total RNA run on a 1% agarose gel, which revealed sharp 28S and 18S rRNA bands with no visible smearing, then resuspended in water treated with diethyl pyrocarbonate (DEPC) after the DNA residues were digested with one unit of RQ1 RNAse-free DNAse. The amount was quantified at 260 nm (Linjawi et al. 2017). A RevertAid™ First Strand cDNA Synthesis Kit was used to reverse transcribe 20 µL of complete poly (A)^+^ RNA extracted from the MCF-7 cell line. The RT reaction was run for 10 min at 25 °C, then for 1 h at 42 °C, and for 5 min at 99 °C as a denaturation step. The StepOne™ Real-Time PCR System (Thermo Fisher Scientific, Waltham, MA, USA) was used to determine the cDNA copy number of the MCF-7 cell lines. The designed primer sequences for the cancer-associated genes associated with breast cancer cell lines (Bcl-2, BAX, and p53 genes) are listed in Table S2 (Brito et al. 2013; Khalil et al. 2019). The 2 − ΔΔCT approach was utilized to ascertain the target’s relative quantification in relation to the reference (Ramadan et al. 2019; Refaie et al. 2020).

### L-asparaginase’s in vivo cytotoxicity in mice

Six- to eight-week-old Swiss albino female mice weighing approximately 40 g were used in the experiment. The mice were graciously donated by the Veterinary Teaching Hospital at Assiut University, Assiut Governorate, Egypt, where the experiment was conducted. Among the mice, there were two groups, each with ten mice. The first group was given 1X phosphate-buffered saline as a control. Two times a week, 500 IU/kg *B. paralicheniformis* AUMC B-516’ L-asparaginase was injected into the second group. Seven weeks were dedicated to the experiment. Blood samples were obtained 15, 30, and 45 days after the final injection. The injections were administered through the tail vein, and blood collection was performed via retro-orbital bleeding to obtain the required sample volumes. After extraction, the serum and plasma were stored at–40 °C. The complete blood count and the levels of transaminases, albumin, total protein, urea, creatinine, and alkaline phosphatase were determined (El-Naggar et al. 2018; Rodrigues et al. 2020). Using universal biochemical keys, the measurements were carried out in the Clinical Chemistry and Hematology laboratories of the Clinical Pathology Department, South Egypt Cancer Institute, Assiut University, Assiut, Egypt, and served as toxicity markers.

### Statistical analysis

The mean and standard deviation (SD) of the tentative study performed in triplicate were used to express all the data. Analysis of the statistical significance was conducted (Stahle and Wold 1989). Differences were deemed significant at *p* ≤ 0.05.

## Results

### Molecular identification of the potent bacterial isolate

**Fig. 1:**
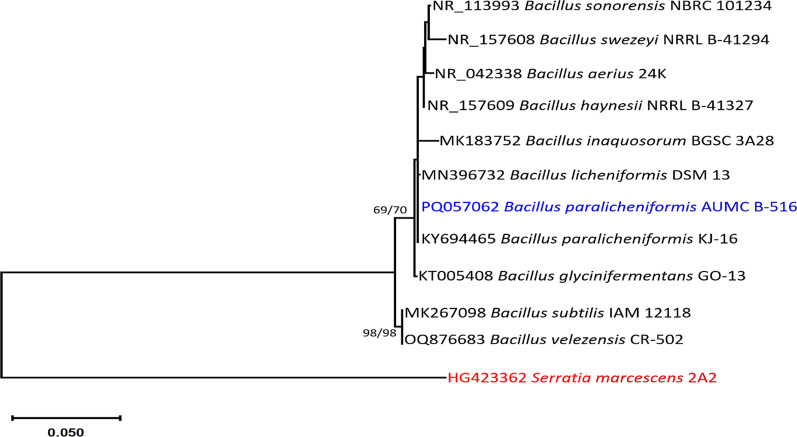
The most parsimonious phylogenetic tree generated from ML/MP analysis using a heuristic search (1000 replications) of 16S sequence of *B. paralicheniformis* AUMC B-516 (in blue) compared to other closely similar species to the genus *Bacillus* in GenBank

On the basis of a megablast search in the NCBI database utilizing the 16S sequence of the bacterial isolate AUMC B-516 in this investigation, the closest hit was *Bacillus licheniformis* strain QT331 [(GenBank accession number MT043736; identities = 1415/1417 (99.86%); gaps = 1/1417 (0%)]. Compared with the type materials, the most comparable species was the *B. paralicheniformis* strain KJ-16 [GenBank accession number KY694465; identities = 1413/1415 (99.86%)]. The phylogenetic study included 12 sequences that yielded 1419 characters when aligned. Among these characters, 979 were perfectly aligned (no gaps, no N), 243 were considered variable (24.8% complete), and 24 were considered informative (2.5% complete). The maximum parsimony analysis produced 7 phylogenetic trees, the most parsimonious of which may show the connection between taxa. The phylogenetic tree had a length of 276, the greatest log likelihood was − 3108.83, the consistency index was 0.740000, the retention index was 0.771930, and the composite index was 0.571228 (Fig. 1). The bacterial isolate in this study was located on the same branch as the *B. paralicheniformis* strain KJ-16. As a result, it was identified here as *B. paralicheniformis*, and its 16S sequence has been uploaded to GenBank as PQ057062 (Fig. 1).

### Optimization of L-asparaginase production parameters

**Fig. 2:**
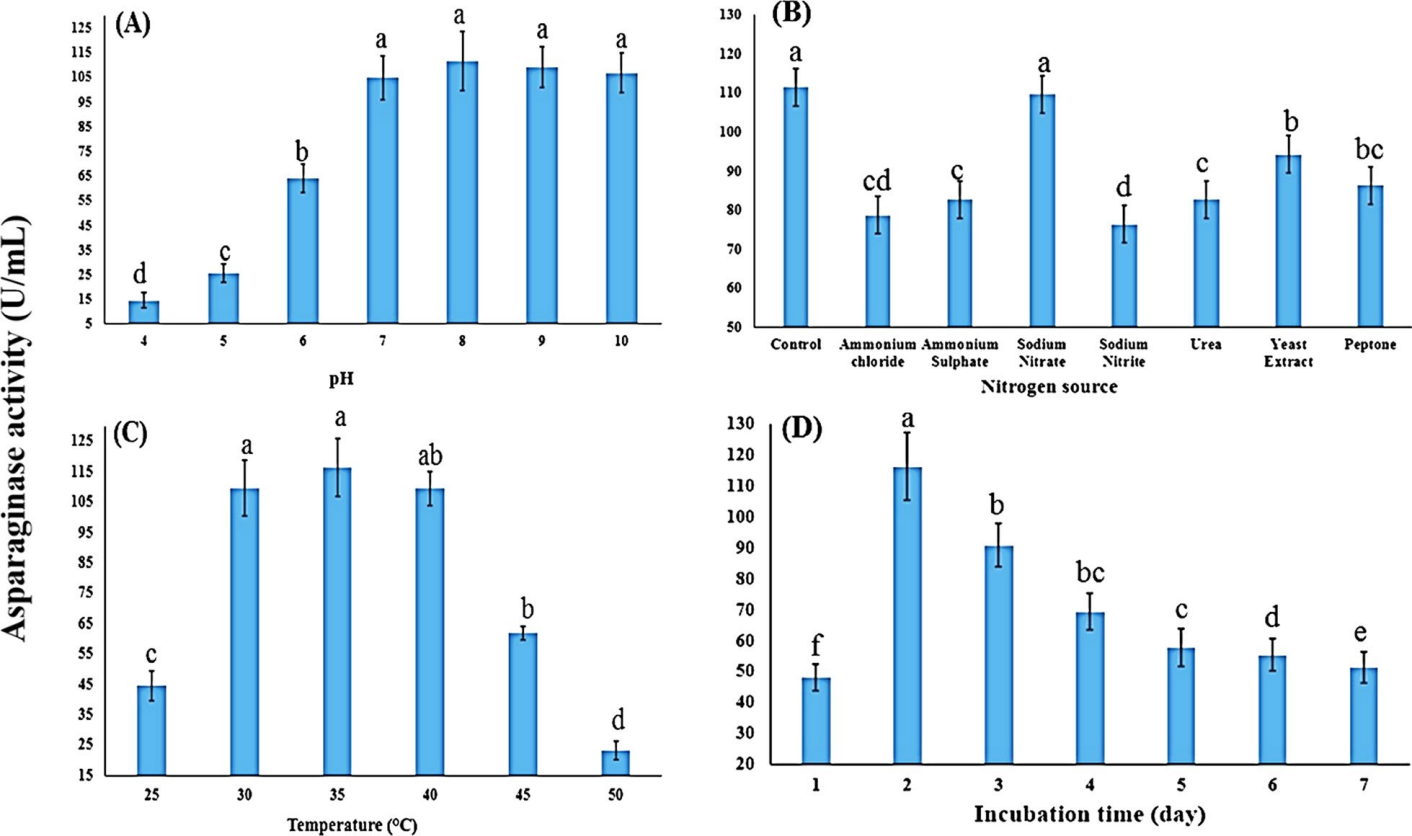
Impact of fermentation factors on asparaginase activity generated by *B. paralicheniformis* AUMC B-516 in SmF. **A** Medium’s pH. **B** Medium’s nitrogen supply. **C** Incubation temperature. **D** Incubation time

On the basis of the findings of the present study, *B. paralicheniformis* AUMC B-516 presented the highest level of L-asparaginase activity (111.4 ± 12 U/mL) at pH 8.0 (Fig. 2A). L-asparaginase activity decreased when a nitrogen source was added to the fermentation medium; and increased when all nitrogen sources were removed from the fermentation medium with the exception of L-asparagine, sodium nitrate was the most significantly effective source (*p* < 0.05), resulting in 109.6 ± 9.2 U/mL, This indicates that no additional nitrogen sources need to be added other than L-asparagine when compared to control value (111.4 ± 12 U/mL) (Fig. 2B).

Regarding temperature, the highest activity was observed at 35 °C, with an activity significantly (*p* < 0.05) increased to of 116.4 U/ml, demonstrating that this temperature promotes maximum enzyme efficiency (Fig. 2C). L-asparaginase activity significantly (*p* < 0.05) increased to 116.4 ± 11 U/mL after 48 h of incubation (Fig. 2D). The alphabets (a, b, c) represent the results of a post-hoc analysis following ANOVA. Groups sharing the same letter are not significantly different from each other, while groups with different letters are statistically significantly different at *p* ≤ 0.05.

### Amino acid analysis

**Fig. 3:**
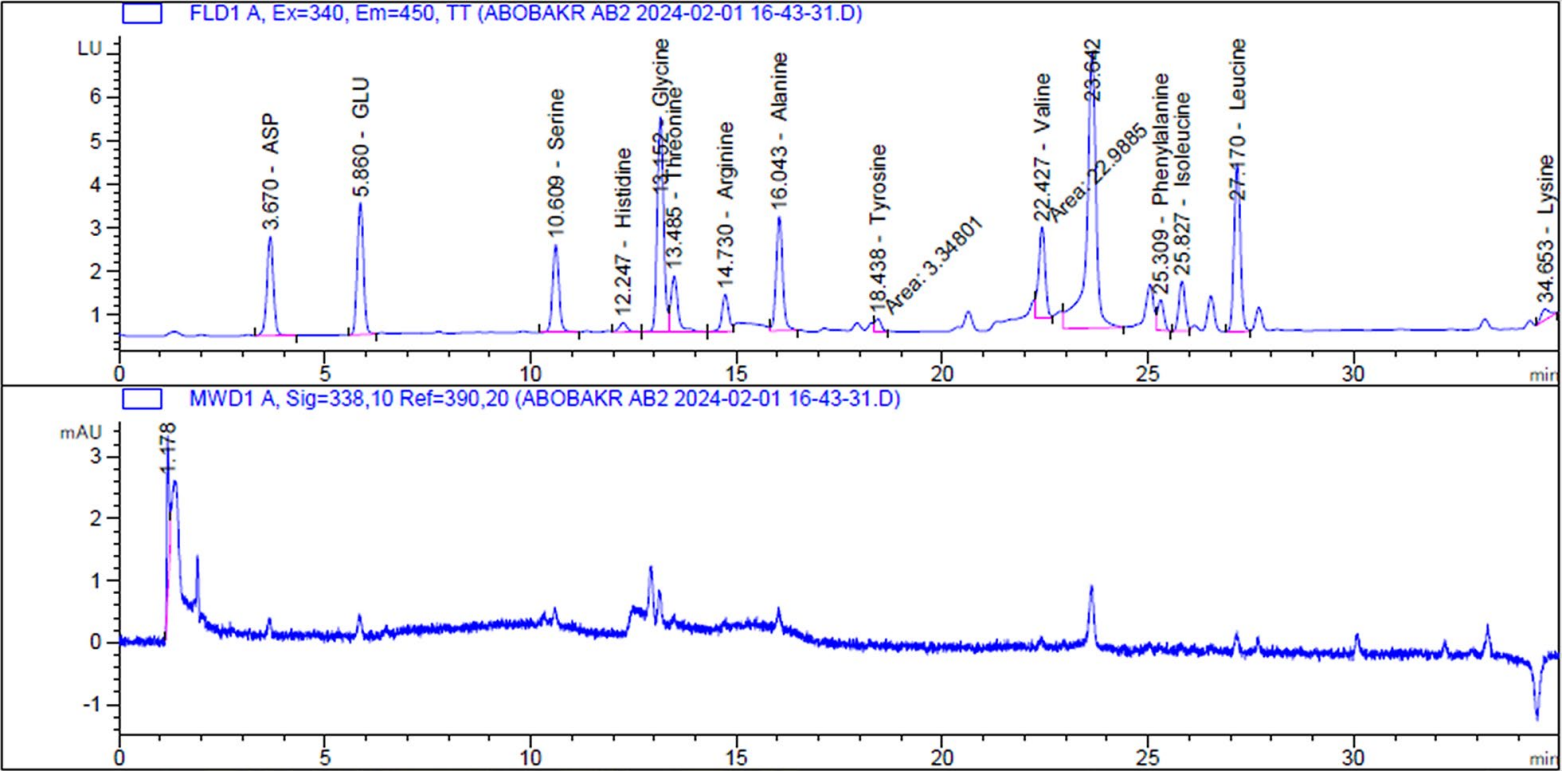
Chromatogram of amino acids profile determined in the crude protein sample produced by *B. paralicheniformis* B-516

Among the crude protein samples that were isolated following *B. paralicheniformis* B-516 fermentation, fifteen amino acids (excluding L-cystine, L-methionine, and L-proline) were identified. The determined amino acids were present at different concentrations, with l-glutamine (1.13 mg/g) being the most abundant, followed closely by L-leucine (0.88 mg/g), L-asparagine (0.75 mg/g), and l-glycine (0.75 mg/g). The remaining amino acid concentrations ranged from 0.12 mg/g for L-tyrosine to 0.48 mg/g for L-alanine (Fig. 3).

### Purification of L-asparaginase

*Bacillus paralicheniformis* AUMC B-516 produced L-asparaginase after 2 days of culture at pH 8.0 and 35 °C without a nitrogen source other than L-asparagine. The DEAE-cellulose gel produced 90 pooled fractions with the greatest number of active L-asparaginase and protein peaks. After the highest-activity fractions from the DEAE-cellulose column were collected, they were further purified via a Sephacryl S-200 HR column. The most active L-asparaginase components isolated from *B. paralicheniformis* AUMC B-516 were purified via Sephacryl S-200 HR to yield two prominent broad peaks of L-asparaginase and protein activity. Following two columns of processing, there was a 12-fold increase in the specific activity of the purified L-asparaginase, resulting in a protein yield of 3.4% and a specific activity of 4087.6 U/mg (Table 1).

**Table 1:** Purification profile of L-asparaginase produced by *B. paralicheniformis* AUMC B-516

Purification steps	Volume (mL)	Activity U/mL	Total activity (U)	Protein mg/mL	Total protein (mg)	Specific Activity (U/mg)	Yield %	Fold
Fermentation media	1278	78.2	99,939.6	0.23	293.94	340	100	1
Ethyl alcohol	195	188.3	36,718.5	0.3512	68.5	536.16	23.3	1.57
DEAE-Cellulose	45	872.3	39,253.5	0.3177	14.3	2745.67	4.86	8.0
Sephacryl S 200 HR	33	1238.13	40,858.5	0.3029	9.996	4087.6	3.4	12.0

### SDS‒PAGE

**Fig. 4:**
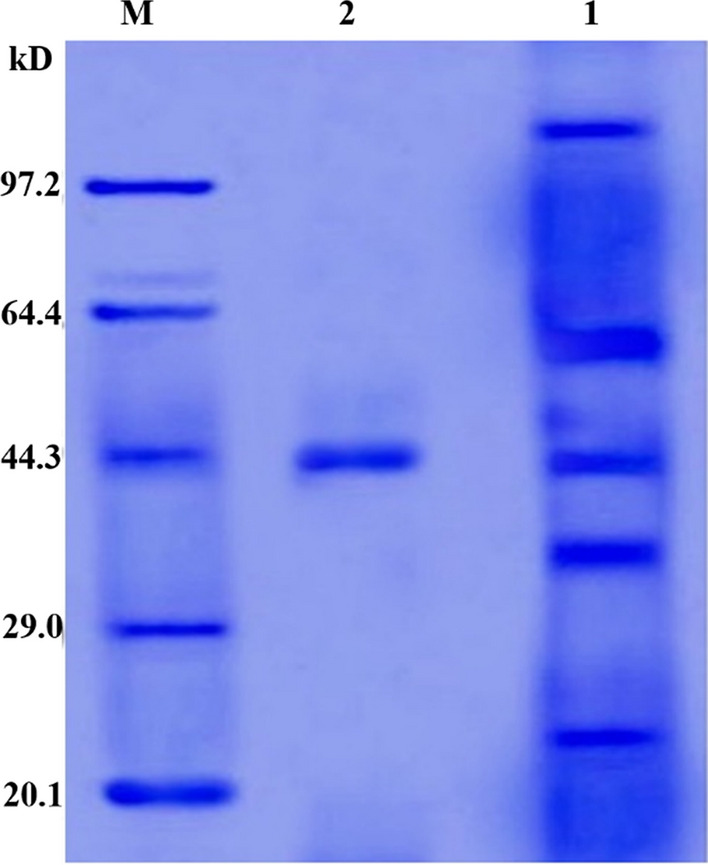
SDS-PAGE of asparaginase produced by *B. paralicheniformis* AUMC B-516. M: pre-stained marker. Lane 1: crude enzyme. Lane 2: pure asparaginase

SDS‒PAGE revealed that the L-asparaginase generated by *B. paralicheniformis* AUMC B-516 was homogenous and completely purified. The calculated molecular weight was 43.98 kDa (Fig. 4).

### The impact of temperature and pH on pure L-asparaginase activity

**Fig. 5:**
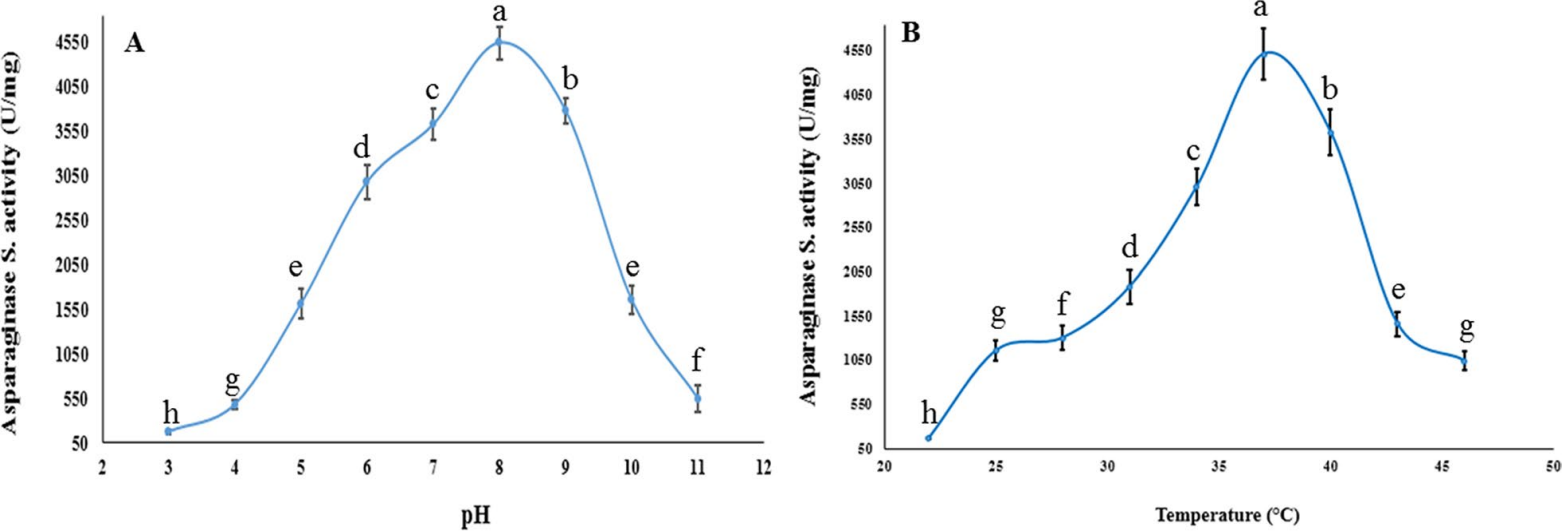
The impact of **A** pH and **B** temperature on the enzymatic activity of the purified L-asparaginase produced by *B. paralicheniformis* AUMC B-516

Pure L-asparaginase showed increased activity throughout a limited pH range, according to the current investigation. (7–8) with pH 8.0 acting as the optimal value showing asparaginase activity peak (4550.15 ± 220 U/mg) at 37 °C (Fig. 5A, B). At pH 9, 10, and 11, the enzyme maintained 83.22, 36.45, and 12.05% of its activity, respectively (Fig. 5).

### Effects of metal ions and inhibitors on *B. paralicheniformis* AUMC B-516’ L-asparaginase

Among all the ions tested, K^+^ had the greatest stimulatory effect, increasing to the top position, followed by Na^+^, which exhibited substantial increases of 118.36 ± 6.5% and 111.27 ± 6.99%, respectively. The activity of L-asparaginase was reduced by the presence of all the other tested ions, with residual activity levels ranging from 7.1 ± 0.5% for Cd^2^⁺ to 94.26 ± 5.2% for Ca^2^⁺, indicating significant variation in the enzyme’s tolerance to different metal ions (Table S3).

### Determination of kinetic parameters (K_m_ and V_max_) and substrate specificity

Using L-asparagine, l-glutamine, aspartic acid, and glutamic acid as substrates at different doses (2–20 mM), K_m_ and V_max_ were calculated. L-asparagine showed the maximum affinity for *B. paralicheniformis* B-516’ L-asparaginase, making it the best suitable substrate, according to the Line Weaver Burk plots. Aspartic acid, glutamic acid, L-asparagine, and l-glutamine were shown to have K_m_ and V_max_ values of 6.22 × 10^−2^ mM and 120.75 µmol/min (Fig. S1), 8.44 mM and 65.82 µmol/min, 9.6 mM and 54.33 µmol/min, and 11.3 mM and 43.60 µmol/min, respectively (Table S4). The enzyme’s affinity for L-asparagine (Km = 6.22 × 10^−2^ mM) is higher than its affinity for l-glutamine (Km = 11.3 mM).

### Molecular docking

Through molecular docking, the expected binding affinity (∆G in kcal/mol) between L-asparagine and L-asparaginase from *B. paralicheniformis* was ascertained. The L-asparaginase of *B. paralicheniformis* had a low ∆G of -3.8 kcal/mol, indicating a high affinity for L-asparagine. Antigenicity scores as anticipated by Vaxijen were also provided. *Bacillus paralicheniformis* L-asparaginase presented a marginally low score of 0.6345, indicating that it was thought to include immune-stimulating epitopes (Table S5).

**Fig. 6:**
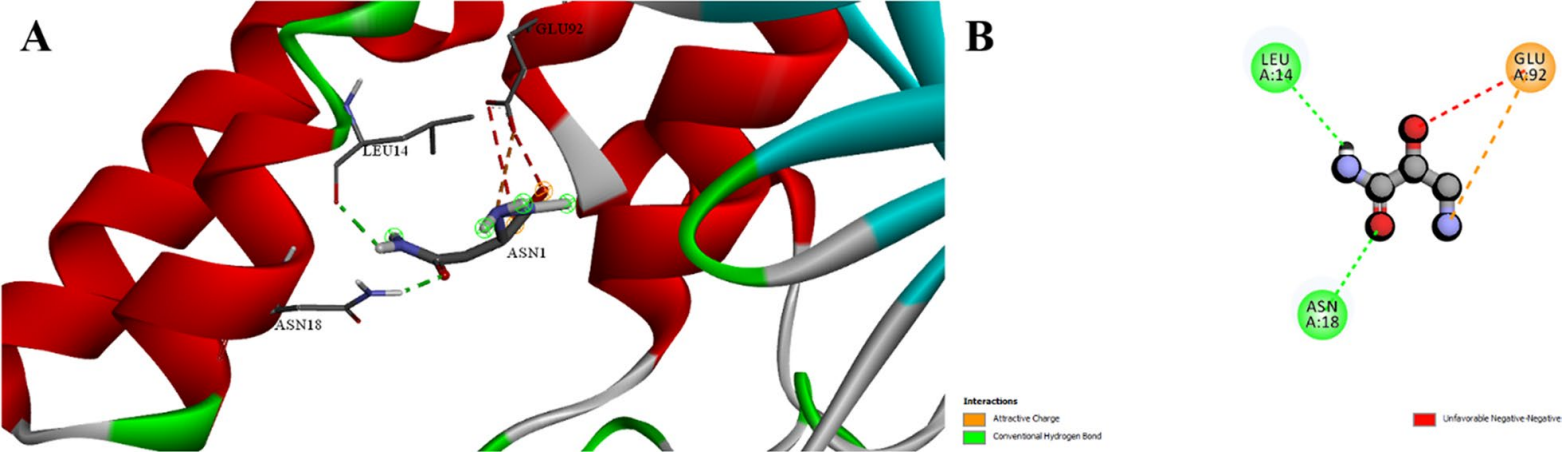
Interaction between asparagine and *B. paralicheniformis*’ asparaginase. **A** 3D, **B** 2D

A greater number of H-bonds with Leu14 and Asn18 were identified via *B. paralicheniformis* L-asparaginase docking, which also predicted the types of interactions and interacting residues. Furthermore, *B. paralicheniformis* interacted favorably with Glu92 but unfavorably with the same residue. *Bacillus paralicheniformis* L-asparaginase had a high affinity, which was probably due to the greater number of H-bonds and more advantageous charged interactions (Fig. 6). With a low score of − 162.08, *B. paralicheniformis* L-asparaginase was evaluated for its activity against the kidney isoform of glutaminase. The *B. paralicheniformis* enzyme (in purple) pierces the binding cavity less deeply in the three-dimensional poses. However, *B. paralicheniformis* L-asparaginase had a more favorable score of − 203 when it was docked to the liver isoform of glutaminase (Fig. S2). These score differences are reflected in the 3D postures, where a larger portion of the binding site is occupied by the *B. paralicheniformis* enzyme. These initial computational findings suggest that *B. paralicheniformis* L-asparaginase is likely more selective for the liver isoform overall. Addressing the inhibition of glutaminase in different tissues may benefit from this specificity.

### Pure L-asparaginase’s cytotoxic impact on MCF-7 human cell lines (in vitro)

**Fig. 7:**
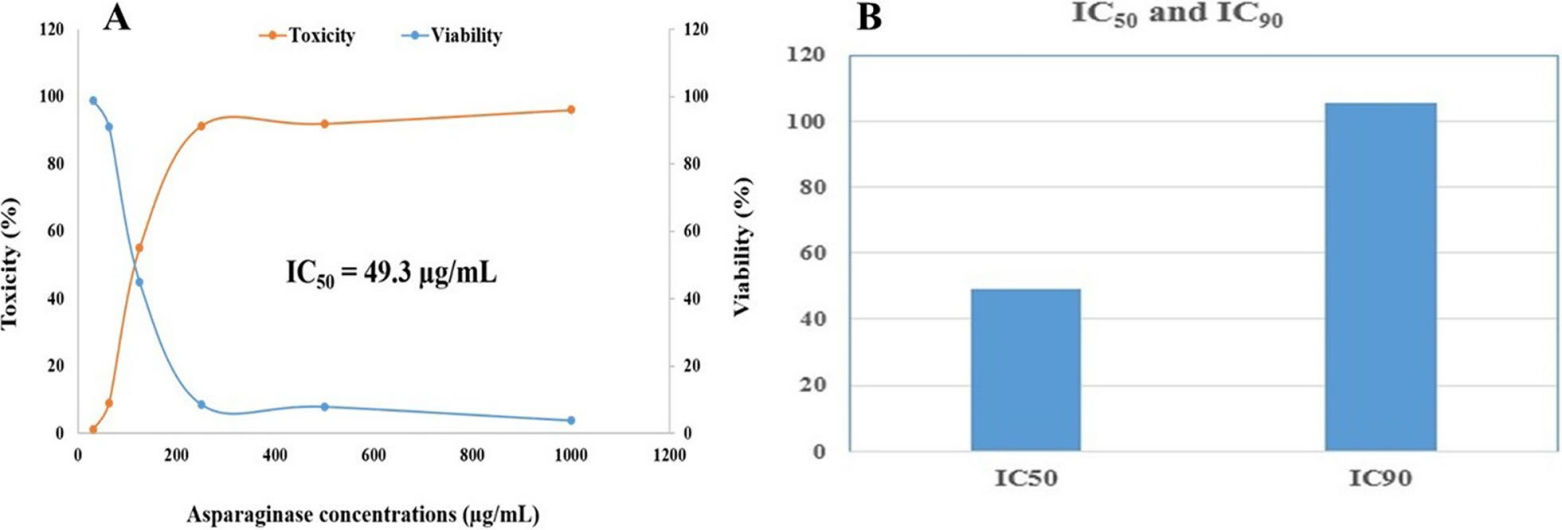
**A** MCF-7 cell lines’ toxicity and viability percentage in relation to the effects of various *B. paralicheniformis* B-516’ asparaginase concentrations, **B** IC_50_ and IC_90_

In this study, untreated MCF-7 cells were used as the negative control (Fig. S3A); MCF-7 cells treated with 100 µg/mL doxorubicin (Fig. S3B) served as the positive control; and MCF-7 cells treated with the IC_50_ of *B. paralicheniformis* B-516’ L-asparaginase (Fig. S3C) were used as the negative control. Cytotoxicity was significantly induced in MCF-7 cells treated with 1000, 500, 250, 125, 62.5, or 31.25 µg/mL *B. paralicheniformis* B-516’ L-asparaginase (Fig. S4), with an IC_50_ of 49.3 µg/mL and an IC_90_ of 105.6 µg/mL (Fig. 7A, B) Both the probability and the observed responses increased as the concentration increased (Fig. S5).

### Flow cytometry results

The flow cytometry analysis of MCF7 cells treated with varying concentrations of asparaginase enzyme (10, 20, 30, 40, and 50 µg/mL) demonstrated a clear and significant dose-dependent increase in the late apoptosis rate. The control group showed a minimal late apoptosis rate of 0.03%, suggesting that untreated MCF7 cells maintain a relatively low level of apoptosis. In the 10 µg/mL treatment group, the apoptosis rate was slightly reduced to 0.02%, indicating that this concentration had little to no effect on late apoptosis.

**Fig. 8:**
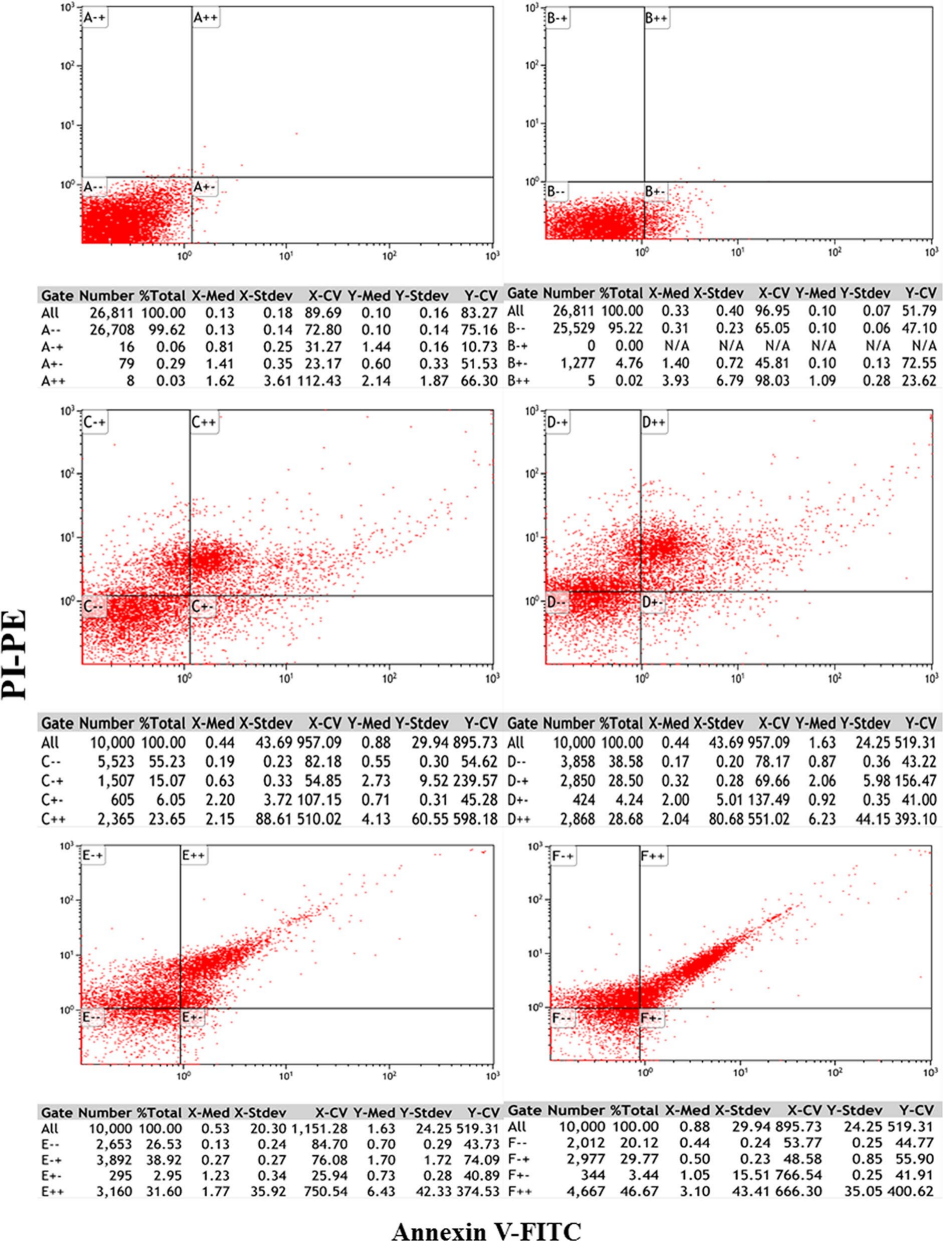
Pure L-asparaginase of *B. paralicheniformis* AUMC B-516 induce apoptosis, **A** Control, **B** MCF-7 cells were treated with 10 μg/mL of enzyme for 48 h, **C** MCF-7 cells were treated with 20 μg/mL of enzyme for 48 h, **D** MCF-7 cells were treated with 30 μg/mL of enzyme for 48 h, **E** MCF-7 cells were treated with 40 μg/mL of enzyme for 48 h, **F** MCF-7 cells were treated with 50 μg/mL of enzyme for 48 h, and apoptosis was determined by flow cytometry using Annexin V/PI double staining

However, at higher concentrations, asparaginase exhibited a marked ability to induce late apoptosis. Specifically, at 20 µg/mL, 23.65% of cells were in late apoptosis, and this increased to 28.68% at 30 µg/mL. Further increases in concentration resulted in even higher apoptosis rates, with 31.60% at 40 µg/mL and a significant 46.67% at 50 µg/mL (Fig. 8). The increase in late apoptosis at higher concentrations suggests a dose-dependent mechanism of action,. The results also imply that asparaginase could be particularly beneficial in targeting cancer cells that are resistant to conventional therapies. Statistical analysis of the apoptosis percentages confirms the significant differences between concentrations. The increase in apoptosis rate from 0.02% at 10 µg/mL to 46.67% at 50 µg/mL is statistically significant, further supporting the hypothesis that asparaginase’s effectiveness increases with dosage. This reinforces its potential as a potent therapeutic agent for inducing cancer cell death, particularly in malignancies where apoptosis resistance is a challenge.

### Gene expression

The MCF-7 cells treated with both *B. paralicheniformis* B-516’ L-asparaginase and doxorubicin showed considerably (*p* < 0.05) lower levels of the antiapoptotic gene BCL-2 than the negative control samples (Fig. S6A). *Bacillus paralicheniformis* B-516’ L-asparaginase and doxorubicin substantially (*p* < 0.05) enhanced the expression of the proapoptotic genes p53 (Fig. S6C) and BAX (Fig. S6B) in MCF-7 cells.

### Fragmenting DNA

**Fig. 9:**
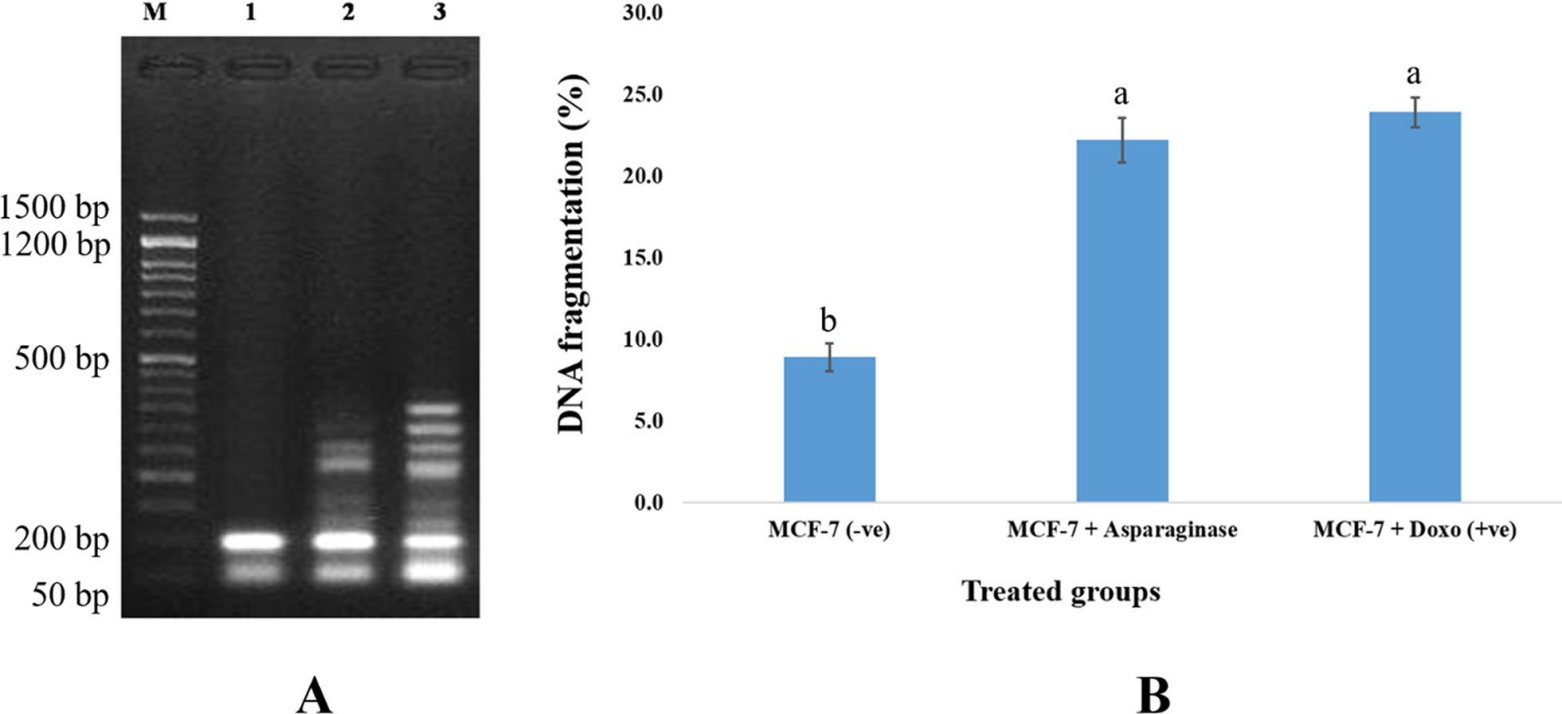
**A** Agarose gel analysis of MCF-7 cancer cell lines exposed to various compounds reveals DNA fragmentation. M: stands for DNA marker; Lanes 1: MCF-7 negative controls; Lane 2: MCF-7 treated with *B. paralicheniformis* B-516’ asparaginase, and Lane 3: MCF-7 treated with Doxorubicin. **B** DNA fragmentation identified in breast cancer cell lines (MCF-7) treated with *B. paralicheniformis* B-516’ asparaginase and Doxorubicin (Means with different figures between treatments in the same column are substantially different at *p* < 0.05)

The results showed that the negative control samples had substantially lower DNA fragmentation rates than the treated samples (*p* < 0.01) (8.9 ± 0.83). In contrast, the DNA fragmentation values in the breast cancer cell line samples treated with *B. paralicheniformis* B-516’ L-asparaginase (22.2 ± 1.36) and the drug doxorubicin (23.9 ± 0.93) were significantly greater (*p* < 0.01) (Fig. 9A&B).

### Pure L-asparaginase’s cytotoxic effects in vivo in an animal model

There were no impacts on glucose, other electrolytes, the liver, or the kidneys, according to the biochemical profiles. On the other hand, the total bilirubin level and the activities of aspartate aminotransferase (AST) and alanine transaminase (ALT) were slightly elevated. These results suggested that *B. paralicheniformis* B-516’ L-asparaginase had relatively little effect on liver function (liver impairment was most likely indicated by AST and ALT markers). All the hematological parameters were within normal ranges during the experiment; however, compared with those in the control group (preinjection), the white blood cell (WBC), platelet, hemoglobin, and red blood cell counts were slightly lower after the injection of L-asparaginase for 15 days. Furthermore, the rats were alive throughout the study periods (Tables S6—S8).

## Discussion

Cross-reactivity with l-glutamine and urea has been demonstrated by commercially available formulations of *E. chrysanthemi* and *E. coli* L-asparaginases, which are thought to be the best for clinical applications (Ghasemi et al. 2017). Therefore, there is a need to investigate new bacterial sources of L-asparaginase. Thus, the goal of the current investigation was to identify an additional microbial source of L-asparaginase. In this study, *B. paralicheniformis* AUMC B-516, which was improved, purified, and used as an anticancer medication, was used to produce L-asparaginase.

By maximizing L-asparaginase synthesis by *B. paralicheniformis* AUMC B-516 in this study, it may be possible to better understand how the independent factors affect L-asparaginase production and to identify the maximum activity levels and fermentation parameters. The highest L-asparaginase activity (78.2 U/mL) was produced by *B. paralicheniformis* AUMC B-516 after 48 h at pH 8.0 and 37 °C when 0.2% glucose and 1.0% L-asparagine were used. Microorganisms differ in their nutritional requirements, which are crucial for achieving maximum L-asparaginase synthesis. In this context, glucose serves as the optimal carbon source for *B. licheniformis* to produce L-asparaginase (Mahajan et al. 2012).

By varying the way that components flow from the cell membrane, the pH and temperature of the growth medium affect the production of enzymes (Castro et al. 2021). Our results revealed that the ideal pH for L-asparaginase production from *B. paralicheniformis* was 8.0, in contrast to the findings of Alrumman et al. (Alrumman et al. 2019), who demonstrated that the optimal pH for L-asparaginase production from *B. licheniformis* was 6.5. By combining tryptone and yeast extract with 0.1% lactose and 0.2% L-asparagine, L-asparaginase can be produced from *E. coli* K-12 (Vimal and Kumar 2017). *Bacillus australimaris* produces L-asparaginase at optimal levels on M9 media supplemented with 2.5% L-asparagine (Chakravarty et al. 2021). At 30 °C, pH 8.0, with 0.2% glucose and 0.5% ammonium sulfate, the ideal conditions for maximum synthesis of L-asparaginase under SSF by *Fusarium solani* AUMC 8615 were reached on the fifth day of incubation (Isaac and Abu-Tahon 2016). L-asparaginase purified from marine derived *Aspergillus flavus* showed stable activity between pH 7.0 and 8.0 (Nurçe et al. 2023).

The maximum L-asparaginase activity of 12.83 U/mL was obtained after 7 days of incubation at pH 8.0 and 27.5 °C (Parashiva et al. 2023). In M9 medium containing 0.2% sucrose and 1.0% L-asparagine, *Bacillus halotolerans* ASN9 generated the highest amount of L-asparaginase (9.25 U/mL) at pH 6.0 and 37 °C, with a specific activity of 256 U/mg (Shafqat et al. 2023b). *Escherichia coli* was able to express *Phaseolus vulgaris* L-asparaginase in large yields, with maximum activity at pH 9.0 and 40 °C (da Silva Gomes et al. 2024). Our findings differ from the optimum submerged medium ingredients that have been previously reported. All of these findings point to the distinct development needs of the strains under investigation, which support their best L-asparaginase production. The biotechnological importance of our strain is established by its production of a substantial amount of extracellular L-asparaginase and its less complicated and expensive growth needs. The conclusions of this study regarding the best fermentation conditions to maximize the synthesis of L-asparaginase provide important new information about the scalability of the process. The improved conditions observed in our findings may result in a more economical and high-yield synthesis of L-asparaginase. This feature is essential for large-scale enzyme application, especially in the pharmaceutical sector, where yields and manufacturing costs play crucial roles.

In this study, the amino acid profile of the crude L-asparaginase from *B. paralicheniformis* B-516 was examined. The amino acid composition was determined to be fifteen (methionine, proline, and cysteine were excluded). l-glutamine was the most prevalent amino acid, followed by leucine, L-asparaginase, and glycine. The identified amino acids were discovered in various amounts. The tyrosine concentration was 0.12 mg/g, whereas the alanine concentration was 0.48 mg/g. To the best of our knowledge, no studies have investigated the amino acid composition of L-asparaginases produced by microorganisms. As a result, this research study is the first in this particular area. However, there are still several limitations in our study, while we performed amino acid analysis on the crude sample, further complementary tests are required to provide a more comprehensive understanding of its composition. Additional assessments, such as peptide mapping, mass spectrometry analysis, and functional assays, will be essential to identify the bioactive peptides and fully characterize the protein content. These studies will help in elucidating the potential bioactivity and mechanisms of action of the crude sample, which is beyond the scope of the current analysis.

The L-asparaginase of *B. paralicheniformis* was purified in this study via two chromatography columns, DEAE-cellulose and Sephacryl S 200 HR, resulting in a 12-fold pure enzyme with 3.4% yield and a maximal specific activity of 4087.6 U/mg, which was greater than that reported in several published studies. The current study illustrated a successful purification technique that produced highly active and pure enzymes. In light of this, L-asparaginase produced from *P. carotovorum* has been purified 9.38 times, producing 23.5% and a maximal specific activity of 202.6 U/mg (Do et al. 2019). *Bacillus halotolerans* ASN9 produces L-asparaginase, which has a 24% yield and 3083 U/mg specific activity after 12 purifications (Shafqat et al. 2023b). *Fusarium foetens* L-asparaginase was purified 15.6 times via DEAE-cellulose column chromatography, yielding 39.89% and 231.38 U/mg specific activity (Parashiva et al. 2023). Using a DEAE-cellulose chromatography column, L-asparaginase from *B. licheniformis* PPD37 was purified 12.47 times, yielding an 11.84% yield and 7707 U/mg (Patel et al. 2023). L-asparaginase extracted from *Fusarium equiseti* AHMF4 was purified 2.67 times with a 48% yield and 488.1 U/mg specific activity using anion exchange QFF and Sephacryl S200 columns (El-Gendy et al. 2021). In this investigation, it is possible that the dual purification stages helped achieve improved purity, which is important for therapeutic applications where contaminants might have negative consequences. This study can be considered a major addition to the improvement of purification procedures for medicinal enzymes by examining the purification yields and enzyme recovery rates.

In the present study, the kinetic parameters (K_m_ and V_max_) of the L-asparaginase produced by *B. paralicheniformis* AUMC B-516 were ascertained for L-asparagine, l-glutamine, aspartic acid, and l-glutamic acid. The highest affinity (K_m_ 6.22 × 10^−2^ mg/mL and V_max_ 120.75 μmol/min) was displayed for L-asparagine, the most appropriate substrate. The K_m_ and V_max_ values for l-glutamine (8.44 mg/mL and 65.82 μmol/min), L-aspartic acid (9.6 mg/mL and 54.33 μmol/min), and l-glutamic acid (11.3 mg/mL and 43.6 μmol/min) were also determined for *B. paralicheniformis* B-516’ L-asparaginase. For asparaginases generated by many bacterial strains, K_m_ and V_max_ have been identified. With L-asparagine as a substrate, El-Naggar et al. (El-Naggar et al. 2018) reported that a K_m_ of 2.139 × 10^−3^ M and a V_max_ of 152.6 U/mL/min were observed for L-asparaginase from *Streptomyces brollosae* NEAE-115. The L-asparaginase from *Weissella paramesenteroides* MN2C2 had km and Vmax values of 4.41 mM and 130.72 U/mL/min, respectively (Amer et al. 2022). Shafqat et al. (2023a) calculated the K_m_ and V_max_ for the L-asparaginase of *B. licheniformis* ASN51’ to be 0.04 mM and 7750 U, respectively, whereas *Pseudomonas* sp. PCH199’s isolated L-asparaginase was shown to have K_m_ and V_max_ values of 0.164 mM and 54.78 U/mg, respectively (Darnal et al. 2023). *Brevibacillus borstelensis* ML12’ L-asparaginase was found to have K_m_ and V_max_ values of 0.310 mM and 121.654 µmol/mL/min, respectively (Mukherjee and Bera 2024). The low value of K_m_ obtained from purified L-asparaginase of *S. maltophilia* EMCC2297, denotes the enzyme’s strong affinity for the substrate L-asparagine and is necessary for the targeted elimination of L-asparagine in case of leukemia cells (Sharma and Mishra 2024). Earlier studies for L-asparaginase kinetic parameters had revealed different K_m_ values from different microorganisms which was 0.059 mM for that produced from *Pseudomonas sp.* PCH44 (Kumar et al. 2022) and 9.74 mM for that of *Sarocladium strictum* (Golbabaie et al. 2020).

On the other hand, certain studies have also identified the K_m_ and V_max_ for fungal L-asparaginases. According to Dias et al. (Dias et al. 2016), purified *Aspergillus oryzae* CCT 3940’ L-asparaginase was found to have K_m_ and V_max_ values of 0.66 mmol/L and 313 U/mL, respectively. The K_m_ and V_max_ of the purified L-asparaginase from *Fusarium foetens* were 23.82 mM and 210.3 U/mL, respectively (Parashiva et al. 2023). In general, it is difficult to compare the values of enzyme activity and kinetic parameters between different studies because of slight differences in methodology. Therefore, comparisons should be performed with caution.

In the present study, the impact of certain metal ions on the L-asparaginase activity of *B. paralicheniformis* B-516 was assessed. The inhibitory effects of Ca^2+^, Mg^2+^, Mn^2+^, Fe^2+^, Cu^2+^, Zn^2+^, Co^2+^, Cd^2+^, Ba^2+^, and EDTA were demonstrated when those ions were present at 5 mmol/mL. The impacts of ions, activators, and inhibitors have been assessed in several studies. The L-asparaginase activity of *Streptomyces brollosae* NEAE-115 increased considerably in the presence of Mg^2+^ and peaked in the presence of Mn^2+^ and Co^2+^. Nevertheless, there was a slight decrease in L-asparaginase activity in the presence of Zn^2+^ and Ca^2+^. Furthermore, Ni^2+^, Hg^2+^, Ba^2+^, and Cu^2+^ all function as strong inhibitors (El-Naggar et al. 2018). The addition of 5 mmol/mL MgSO_4_ and MnSO_4_ activated the pure L-asparaginase from *A. oryzae* CCT 3940. L-asparaginase activity was slightly reduced in the presence of FeSO_4_, CuSO_4_, KCl, CaCO_3_, and ZnSO_4_. Furthermore, L-asparaginase activity was inhibited by ZnSO_4_, CuSO_4_, and CaCl_2_, which decreased it to approximately 60% (Dias et al. 2016).

On the other hand, the L-asparaginase activity in this study was mostly stimulated by K^+^, Na^+^, Ca^2+^, and Fe^2+^. It has been shown to be comparable to reports of *Bacillus megaterium* H-1 (Zhang et al. 2015) and *Bacillus megaterium* MG1 (Roy et al. 2019). The activity of the recombinant L-ASNase from *B. subtilis* CH11 was considerably enhanced by K^+^, Ca^2+^, and Mg^2+^ ions (Arredondo-Nuñez et al. 2023). However, some authors reported an inhibitory effect of Mn^2+^ ions (Sanghvi et al. 2016; Feng et al. 2019). The activity of *Pseudomonas sp.* PCH199 extremozyme decreased to 51.6% by K^+^ and was enhanced by 127.5% and 111.6% in the presence of Na^+^ and Ca^2+^, respectively (Darnal et al. 2023). The activation of enzymes by some metal ions may be due to their ability to act as cofactors for binding at the catalytic site of the enzyme, while the suppression by other ions may be attributable to their chelation with sulfhydryl groups of protein structures (Kumar et al. 2022; AlShaikh-Mubarak et al. 2023). The L-asparaginase activity of *Brevibacillus borstelensis* ML12 markedly increased in the presence of 10 mmol/mL CoCl_2_, MnCl_2_, or FeCl3. The asparaginase activity of *B. licheniformis* PPD37 was shown to be slightly reduced in the presence of Zn^2+^ and Hg^2+^ but remained rather stable in the presence of Mn^2+^ (Patel et al. 2023). The L-asparaginase activity of *Brevibacillus borstelensis* ML12 significantly increased in the presence of CoCl2 and KCl, whereas the activity significantly decreased in the presence of CuSO_4_, FeSO_4_, KNO_3_, and NaNO_3_ (Mukherjee and Bera 2024). KCl and NaCl have been shown to stimulate *Penicillium* cyclopium L-asparaginase (El-Refai et al. 2016). While Hg^2+^ reduced *Aspergillus niger* AKV-MKBU L-asparaginase activity to 50%, Mn^2+^ was unable to inhibit enzyme activity (Vala et al. 2018). L-asparaginase activity in *Fusarium equiseti* AHMF4 increased by 5.8% and 43.3%, respectively, following incubation with K^+^ and Mg^2+^ at a concentration of 50 mM; however, its activity decreased by 77.5% and 99.1%, respectively, with Ca^2+^ and Na^+^ at 50 mM. Mn^2+^ and Cu^2+^ had little effect; however, Ba^2+^ at a dose of 10 mM increased enzyme activity by 22.5% (El-Gendy et al. 2021). L-asparaginase from *Fusarium foetens* was enhanced by Mn^2+^, Fe^2+^, and Mg^2+^ (Parashiva et al. 2023). The inhibition of enzyme activity by divalent ions may be due to the chelation of the sulfhydryl groups of L-asparaginase with metal ions (Kumar et al. 2022). While EDTA did not show any impact on enzymatic activity. Similarly, EDTA did not affect L-ASNases of *Pseudomonas sp.* PCH199 (Darnal et al. 2023), *B. megaterium* strain MG1 (AlShaikh-Mubarak et al. 2023).

The results of this investigation indicated that *B. paralicheniformis* L-asparaginase has a low level of activity against l-glutamine and a high specificity for L-asparagine, which is advantageous for lowering the possibility of negative side effects such as neurotoxicity, our results in accordance with (Castro et al. 2021; Van Trimpont et al. 2022; Sisay et al. 2024). This comparison demonstrated the potential benefits of L-asparaginase from *B. paralicheniformis* in clinical settings, where selectivity is essential for reducing off-target effects. This specificity may be helpful in addressing the inhibition of l-glutaminase in various tissues. To further confirm and describe the isoform selectivity reported by these protein‒protein docking experiments, more in-vitro binding and enzymatic investigations are needed. The models also serve as a foundation for the structure-based design of modified selectivity profiles for L-asparaginase variations.

L-asparaginase has demonstrated anticancer properties not only against leukemia but also against solid tumors like breast cancer. For example, L-asparaginase derived from *E. coli* MF-107 induces apoptosis in breast cancer cell lines by activating the mitochondrial pathway mediated by the tumor suppressor protein p53, with an IC_50_ value of 5.70 IU/mL (Shahnazari et al. 2022). Similarly, L-asparaginase extracted from *Vigna unguiculata* exhibits cytotoxic effects against MCF-7 cell lines, with an IC_50_ of 15 μg/mL (Moharib 2018). Furthermore, L-asparaginase produced by *Yarrowia lipolytica* has shown cytotoxic activity against MCF-7 and A549 cell lines, with IC_50_ of 3 and 2 IU/mL, respectively (Mazloum-Ravasan et al. 2021).

The antiproliferative capability of *B. paralicheniformis* B-516’ purified L-asparaginase against the MCF-7 breast cancer cell line was examined in this study. This is important since L-asparaginase has a well-established track record for treating a variety of malignancies. For example, the study of Ali et al. (Ali et al. 2016) demonstrated the effectiveness of L-asparaginase against lymphoblastic leukemia; however, our investigation broadens its applicability to breast cancer, suggesting more extensive therapeutic potential. Furthermore, a comparison between the cytotoxic effects observed in our study and the toxic effects reported by Duval et al. (Duval et al. 2002) highlighted the necessity of additional optimization to minimize side effects. Our investigation of asparaginase variation with possibly fewer hypersensitivity reactions—a known problem with currently available commercial formulations is a noteworthy advance. Asselin and Rizzari (Asselin and Rizzari 2015) emphasized the difficulties associated with hypersensitivity in therapeutic applications; our work addresses these issues by identifying a unique source of L-asparaginase.

We have an intriguing point of comparison because *B. paralicheniformis* was chosen as the microbial source for L-asparaginase synthesis in our investigation. *Escherichia coli* and *E. chrysanthemi* have historically been the main sources of L-asparaginase for therapeutic use (Verma et al. 2007; Müller et al. 2023). These strains have several drawbacks, including low yield and hypersensitivity. Some of these issues are resolved by the introduction of *B. paralicheniformis* AUMC B-516 in this work, as the strain showed encouraging antiproliferative activity and increased enzyme production under ideal circumstances. According to this research, *B. paralicheniformis* may be a better option for large-scale manufacturing than strains that have been employed in the past, possibly outperforming them in terms of yield and effectiveness.

One of the main issues with the therapeutic utilization of L-asparaginase is its cytotoxic effects on noncancerous cells. A fair assessment of the therapeutic potential of the enzyme is given by the study’s focus on its antiproliferative qualities against the MCF-7 breast cancer cell line and evaluation of its cytotoxicity to noncancerous cells. Our investigation indicated that the enzyme from *B. paralicheniformis* AUMC B-516 may offer stronger selectivity toward malignant cells, with significantly less toxicity to healthy cells, than the cytotoxicity profiles described previously (Shrivastava et al. 2015; Gervasoni et al. 2017). This selectivity makes *B. paralicheniformis* L-asparaginase a potentially safer therapeutic alternative and is essential for minimizing adverse effects in clinical applications. It is also possible to compare the potential of *B. paralicheniformis* L-asparaginase as a clinical formulation to that of other formulations now in use, such as those made from *E. chrysanthemi* and *E. coli*. This enzyme is positioned for clinical trials because of the emphasis on decreased immunogenicity and its encouraging antiproliferative efficacy. The increase in L-asparaginase concentration was associated with an increase in the cytotoxicity against malignant cells, indicating that the cytotoxicity was dose-dependent (Sharma and Mishra 2023). Because the deamination of the non-essential amino acid asparagine resulted in a decrease in asparagine pool, the anticancer activity of L-asparaginase revealed the effective death of malignant cell types (Darnal et al. 2023). Many researches had supported the effectiveness of L-asparaginase against leukemic cells, as L-asparaginase isolated from *Melioribacter roseus* has nearly the same IC_50_ (3.0 IU/ml) (Sivakumar et al. 2024) of *S. maltophilia* EMCC2297 L-asparaginase. Various L-asparaginases have different IC_50_ as the one isolated from *Rhodospirillum rubrum* has IC_50_ 1.8 IU/mL (Dobryakova et al. 2023).

In addition to its direct antiproliferative characteristics, L-asparaginase has been reported to possess immunomodulatory features that may impact the immune response during the treatment of cancer. The immune-related adverse effects of L-asparaginase, such as its capacity to cause allergic reactions and impair immunological function, have been covered in studies by Asselin B and Rizzari (Asselin and Rizzari 2015) and Müller et al. (Müller et al. 2023). The *B. paralicheniformis* L-asparaginase in this work may have a relatively low immunogenic profile, which could decrease the likelihood of unfavorable immunological responses, according to the current study’s investigation of the features of this enzyme. To evaluate the safety and effectiveness of this enzyme in individuals with a weakened immune system, more studies should be performed to clarify its immunomodulatory effects and compare them with those of formulations that are sold commercially.

## Conclusions

The synthesis of L-asparaginase from *B. paralicheniformis* AUMC B-516 was optimized in this study. The enzyme was purified after being run through two columns for chromatography. The two most stimulating elements were K^+^ and Na^+^. The km and Vmax values of pure L-asparaginase for L-asparagine, l-glutamine, L-aspartic acid, and l-glutamic acid were determined. The molar Gibbs free energy (∆G) and antigenicity values of pure L-asparaginase on L-asparagine were ascertained from the molecular docking results. The DNA fragmentation values of MCF-7 cells treated with purified L-asparaginase from *B. paralicheniformis* B-516 and doxorubicin were also evaluated. The IC_50_ was determined after MCF-7 cells were treated with various doses of *B. paralicheniformis* AUMC B-516’ pure L-asparaginase, which showed significant cytotoxicity. The biochemical profiles of pure L-asparaginase revealed no changes in glucose, other electrolytes, the liver, or the kidneys, although there was a minor elevation in the total bilirubin level and an increase in the activities of aspartate aminotransferase (AST) and alanine transaminase (ALT). Additionally, AST and ALT markers revealed that pure L-asparaginase may have a minor effect on liver function.

## Supplementary Information

Additional file1 (PDF 4463 KB)

## Data Availability

No datasets were generated or analysed during the current study.
