# Supplementary material for: Enhanced production and purification of L-asparaginase from Bacillus paralicheniformis AUMC B-516 with potent cytotoxicity against MCF-7 cell lines
Source: AMB Express. 2025 May 22;15:80. doi: 10.1186/s13568-025-01890-w (PMC12098240; doi:10.1186/s13568-025-01890-w)
Supplement: Supplementary file 1 — Additional file1 (PDF 4463 KB) [file 13568_2025_1890_MOESM1_ESM.pdf]

Enhanced production and purification of L-asparaginase from *Bacillus paralicheniformis* AUMC B-516 with potent cytotoxicity against MCF-7 cell lines

Abdullah Abobakr Saleh[1,2*], Hamdy M. El-Aref [1,3], Azza M. Ezzeldin[4,5], Rania M. Ewida[6], Osama A.M. Al-Bedak[7]

[1]Molecular Biology Researches & Studies Institute, Assiut University, Egypt; (A.A.S.) abdullah.abobakr@science.aun.edu.eg (0000-0002-8597-3842)

[2]South Egypt Cancer Institute, Department of Clinical Pathology and Hematological Malignancies, Assiut University, 71511, Egypt.

[3]Department of Genetics, Faculty of Agriculture, Assiut University, Assiut 71511, Egypt; (H.M.E.) hmelaref_2016@aun.edu.eg (0000-0002-4167-6221)

[4]School of applied health sciences, Badr University Assiut, Assiut, Egypt; (A.M.E.) azza.mezzeldin@bua.edu.eg

[5]Clinical Pathology Department, Faculty of Medicine, Assiut University, 71511, Egypt.

[6]Food Hygiene, Safety and Technology Department, Faculty of Veterinary Medicine, New Valley University, El-Kharga, 72511, Egypt; (R.M.E.) r_ewida@vet.nvu.edu.eg (0000-0003-2110-6851)

[7]Assiut University Mycological Centre, Assiut University, Assiut 71511, Egypt; (O.A.M.A.) osamaalbedak@aun.edu.eg (0000-0003-0465-619X).

[*]Corresponding author: abdullah.abobakr@science.aun.edu.eg

Table S1 Standard Deviation for L-Asparagine concentration and Velocity

Conc. (mM)	Velocity (µmol/min)	Standard Deviation (SD)
2	117.11	±0.025
4	118.9	±0.01
6	119.51	±0.05
8	119.82	±0.046
10	120.0	±0.1
12	120.13	±0.011
14	120.22	±0.026
16	120.28	±0.026
18	120.33	±0.03
20	120.38	±0.03

Table S2 Sequences of primers used for qRT–PCR of the MCF-7 breast cancer cell lines.

**Gene**	**GenBank (accession no.)**	**Primer sequence**
Bcl-2	M14745.1	F: CCT CGC TGC ACA AAT ACT CC<br>R: TGG AGA GAA TGT TGG CGT CT
BAX	XM_054373112.1	F: CTG TAT GTG GGA CTG GTG GT<br>R: GGA AAT GAG GGG TGG AAG GA
p53	X60020.1	F: TGG CCA TCT ACA AGC AGT CA<br>R: GGT ACA GTC AGA GCC AAC CT
GAPDH	AK026525.1	F: CAC ATC GCT CAG ACA CCA TG<br>R: TGA CGG TGC CAT GGA ATT TG

BCL-2: B-cell lymphoma-2 gene; BAX: Bcl-2-associated X protein encoding gene; p53: tumor suppressor gene; GAPDH: housekeeping gene.

Table S3 Effects of metal ions and EDTA (5 mM) on the activity of pure L-asparaginase produced by *B. paralicheniformis* AUMC B-516 (mean±SD, n = 3). The relative activity (%) results are expressed as the proportion of the activity under the tested inhibitory conditions relative to the L-asparaginase activity in the control without inhibitors.

Metal ions	Specific activity (U/mg)	Relative activity (%)
Control	4550.38±248.5[b]	100±5.49[b]
Na^{+}	5063.08±320[a]	111.27±6.99[a]
K^{+}	5385.58±296.5[a]	118.36±6.5[a]
Ca^{2+}	4288.75±237.5[bc]	94.26±5.2[bc]
Mg^{2+}	2386.50±197.5[e]	52.44±4.3[e]
Mn^{2+}	1806.33±117.5[f]	39.69±2.6[f]
Cu^{2+}	645.67±49[h]	14.17±0.99[h]
Fe^{2+}	4161.08±326.5[c]	91.43±7.19[c]
Zn^{2+}	1386.58±107.5[g]	30.47±2.4[g]
Co^{2+}	2006.32±177.5[f]	44.1±3.9[f]
Cd^{2+}	322.17±24[h]	7.1±0.5[h]
Ba^{2+}	1312.19±114[g]	28.84±2.5[g]
EDTA	3225±278[d]	70.87±6.1[d]

Table S4 Kinetic parameters related to the substrate specificity of L-asparaginase

**Substrate**	**V_{max} (µmol/min)**	**K_m (mM)**
L-asparagine	120.75	6.22×10^{-2}
L-glutamine	65.82	8.44
L-aspartic acid	54.33	9.6
L-glutamic acid	43.6	11.3

Table S5 ΔG (kcal/mol) for II L-asparaginase docking with L-asparagine

**Protein**	**ΔG(Kcal/mol)**	**Antigenicity**
B. paralicheniformis' L-asparaginase	-3.8	0.6345

Table S6. Difference in Chemical and Hematological parameters between the studied group and control group after 15 days.

Variables	Control (Mean±SD)	Tested group (Mean±SD)	*P* value
Total proteins	7.75±0.15	6.59±0.3	**< 0.001****
Albumin	3.4±0.15	3.15±0.48	**< 0.001****
Alkaline phosphatase	191.67±5.5	185.4±11.08	0.405^{NS}
Serum GOT (AST)	174.67±6.03	211.6±7.92	**< 0.001****
Serum GPT (ALT)	73.33±3.21	134±9.69	**< 0.001****
Bilirubin total	0.81±0.006	0.94±0.039	**0.001****
Bilirubin direct	0.073±0.006	0.082±0.0084	0.169^{NS}
Urea	51.67±0.58	51.4±0.55	0.537^{NS}
Creatinine	0.96±0.06	0.84±0.13	0.210^{NS}
Serum glucose	93.67±6.66	95.4±7.3	0.749^{NS}
RBCs	$(8.23\times10^{12}) \pm (2.52\times10^{11})$	$(7.56\times10^{12}) \pm (3.89\times10^{11})$	**0.023***
WBCs	$(8.73\times10^{9}) \pm (2.08\times10^{8})$	$(8.22\times10^{9}) \pm (1.17\times10^{9})$	0.493^{NS}
Hemoglobin	14.43±0.06	4.8±1.38	0.671^{NS}
Platelets	$(7.78\times10^{11}) \pm (2.05\times10^{10})$	$(7.37\times10^{11}) \pm (4.53\times10^{10})$	0.201^{NS}

Data expressed as Mean±SD. (*) = $p < 0.05$, (**) = $p < 0.01$, and (NS) = non-significant.

Table S7. Difference in chemical and Hematological parameters between the studied group and control group after 30 days.

Variables	Control (Mean±SD)	Tested group (Mean±SD)	*P* value
Total proteins	7.75±0.12	7.24±0.082	**<0.001****
Albumin	3.44±0.075	3.66±0.031	**<0.001****
Alkaline phosphatase	198.33±4.04	207.22±3.42	**0.016***
Serum GOT (AST)	185±3.6	230±7.84	**<0.001****
Serum GPT (ALT)	73.67±3.21	172.2±6.42	**<0.001****
Bilirubin total	0.87±0.01	0.96±0.031	**0.003****
Bilirubin direct	0.083±0.006	0.084±0.0055	0.875^{NS}
Urea	52±1	52.6±1.14	0.482^{NS}
Creatinine	0.9±0.021	0.93±0.095	0.634^{NS}
Serum glucose	95.33±7.37	98±5.7	0.584^{NS}
RBCs	$(8.03\times10^{12}) \pm (5.03\times10^{11})$	$(8.1\times10^{12}) \pm (1.1\times10^{11})$	0.840^{NS}
WBCs	$(9.1\times10^{9}) \pm (1.53\times10^{8})$	$(9.1\times10^{9}) \pm (1.21\times10^{9})$	0.993^{NS}
Hemoglobin	14.76±0.06	14.9±0.78	0.784^{NS}
Platelets	$(7.53\times10^{11}) \pm (4.06\times10^{10})$	$(7.84\times10^{11}) \pm (9.84\times10^{9})$	0.134^{NS}

Data expressed as Mean±SD. (*) = $p < 0.05$, (**) = $p < 0.01$, and (NS) = non-significant.

Table S8. Difference in Chemical and Hematological parameters between the studied group and control group after 45 days.

Variables	Control (Mean±SD)	Tested group (Mean±SD)	*P* value
Total proteins	7.76±0.15	8.41±0.27	**0.010***
Albumin	3.46±0.05	4.07±0.07	**<0.001****
Alkaline phosphatase	239±4.58	250.2±28.69	**0.539**^{NS}
Serum GOT (AST)	182.33±3.21	265.8±19.06	**<0.001****
Serum GPT (ALT)	78.67±1.15	191.8±7.19	**<0.001****
Bilirubin total	0.88±0.01	0.97±0.02	**<0.001****
Bilirubin direct	0.11±0.01	0.092±0.008	**0.033***
Urea	54.67±1.53	60.4±1.14	**<0.001****
Creatinine	0.99±0.11	0.81±0.41	0.500^{NS}
Serum glucose	91.33±6.66	95.4±6.35	0.421^{NS}
RBCs	$(8.27\times10^{12}) \pm (2.1\times10^{11})$	$(8.64\times10^{12}) \pm (2.41\times10^{11})$	0.068^{NS}
WBCs	$(9.2\times10^{9}) \pm (6.1\times10^{8})$	$(8.94\times10^{9}) \pm (1.11\times10^{9})$	0.684^{NS}
Hemoglobin	15.17±0.31	14.52±1.25	0.427^{NS}
Platelets	$(7.96\times10^{11}) \pm (1.04\times10^{10})$	$(7.61\times10^{11}) \pm (3.49\times10^{10})$	0.152^{NS}

Data expressed as Mean±SD. (*) = $p < 0.05$, (**) = $p < 0.01$, and (NS) = non-significant.

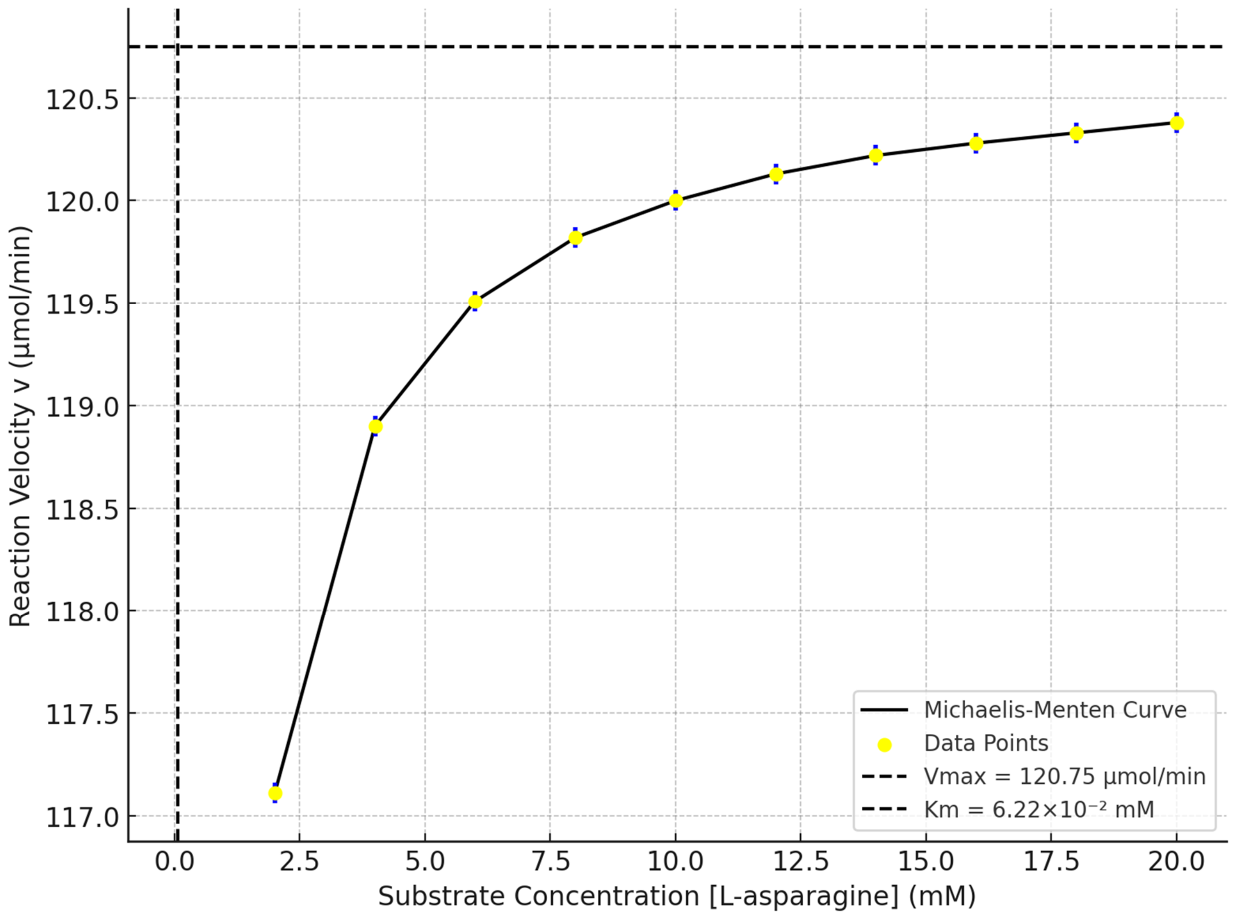

Fig. S1 Michaelis-Menten curve with Km=0.0622, Vmax=120.75 µmol/min (maximum reaction velocity).

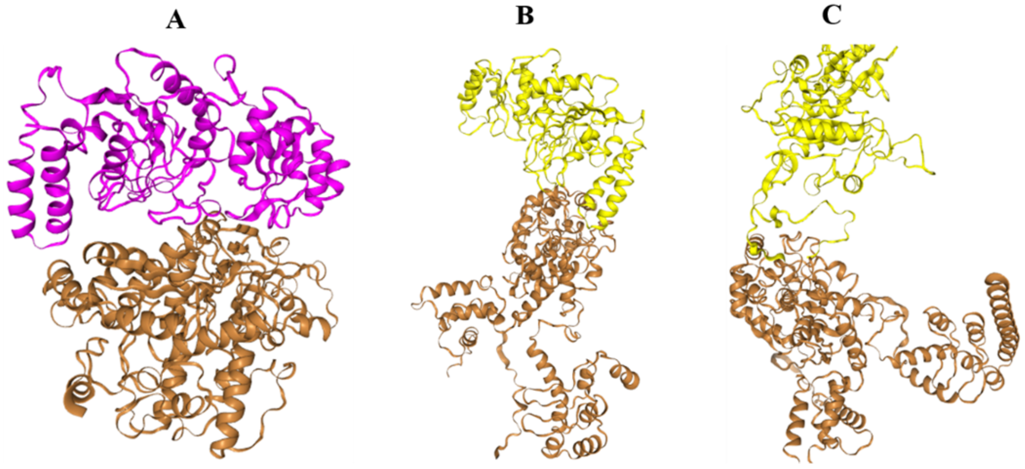

Target	Asparagine	Glutaminase kidney isoform, mitochondrial	Glutaminase liver isoform, mitochondrial
Docking score	-248	-162.08	-203

Fig. S2 Molecular docking score and 3D interaction between **A** *Bacillus paralicheniformis*' asparagine (in purple). **B** glutaminase kidney isoform, mitochondrial. **C** glutaminase liver isoform, mitochondrial.

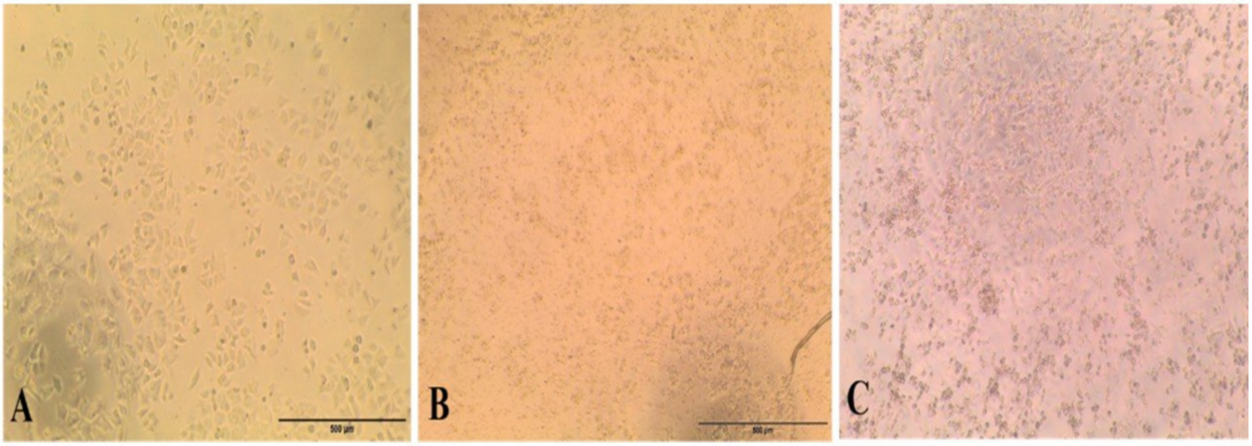

Fig. S3 MCF-7 cell lines. **A** untreated cells (negative control). **B** MCF-7 cell treated with 100 μg/mL of Doxorubicin. **C** MCF-7 treated with *B. paralicheniformis* B-516'asparaginase.

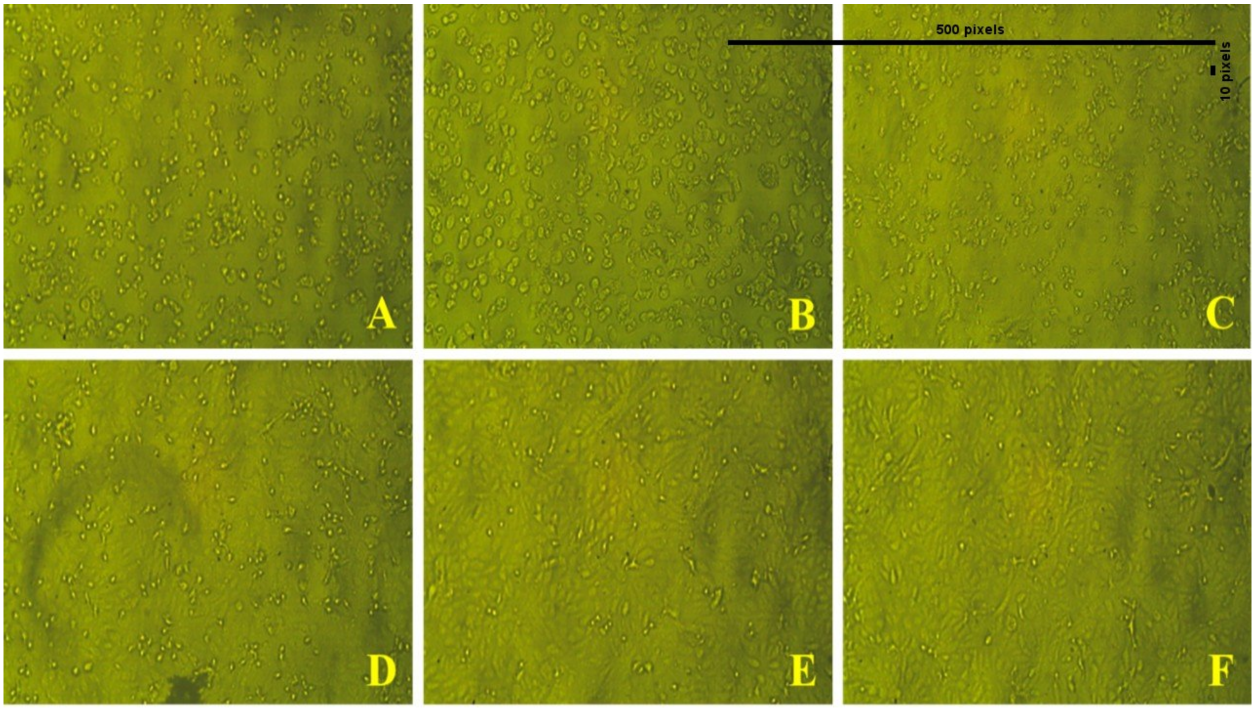

Fig. S4 In-vitro effect of different concentrations. **A-F** 1000, 500, 250, 125, 62.5, and 31.25 µg/mL of *B. paralicheniformis* B-516’asparaginase on MCF-7 cell lines.

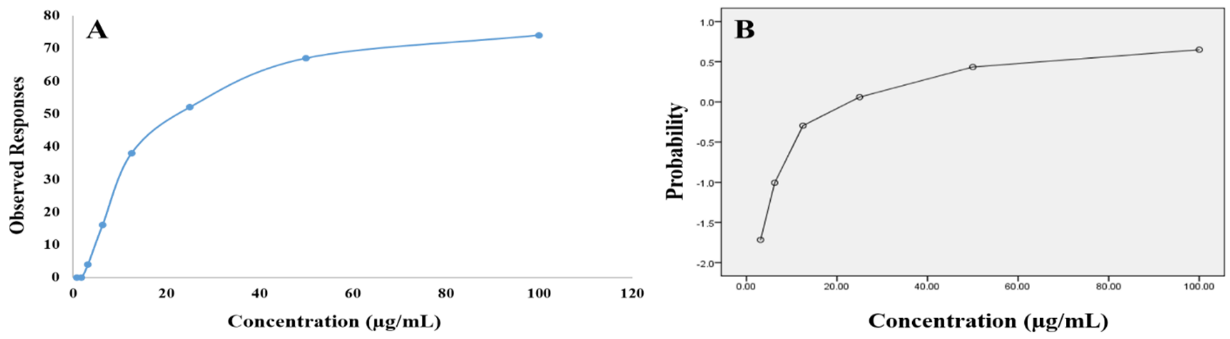

Fig. S5 (**A**) Observed responses. (**B**) Probability. of MCF-7 cell lines death treated with different concentrations of *B. paralicheniformis* B-516' asparaginase.

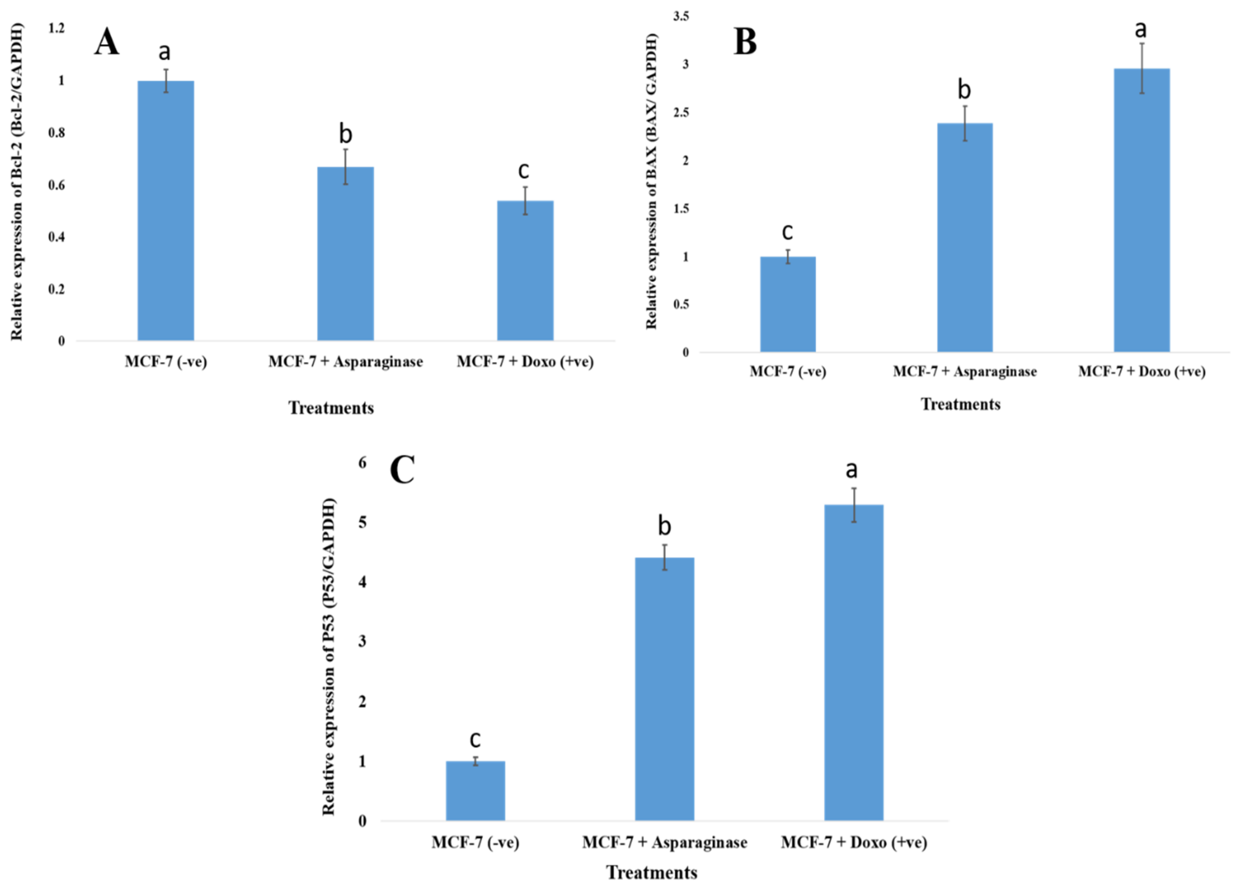

Fig. S6 Gene expression of (**A**) BCL-2. (**B**) BAX. (**C**) p53. in MCF-7 breast cancer cell linestreated with both *B. paralicheniformis* B-516' asparaginase and Doxorubicin medication.
